# Attractor landscape analysis of colorectal tumorigenesis and its reversion

**DOI:** 10.1186/s12918-016-0341-9

**Published:** 2016-10-20

**Authors:** Sung-Hwan Cho, Sang-Min Park, Ho-Sung Lee, Hwang-Yeol Lee, Kwang-Hyun Cho

**Affiliations:** 1 Laboratory for Systems Biology and Bio-Inspired Engineering, Department of Bio and Brain Engineering, Korea Advanced Institute of Science and Technology (KAIST), Daejeon, 34141 Republic of Korea; 2 Graduate School of Medical Science and Engineering, Korea Advanced Institute of Science and Technology (KAIST), Daejeon, 34141 Republic of Korea

**Keywords:** Colorectal tumorigenesis, Attractor landscape analysis, Human signaling network, Cancer reversion, Reverse control, Systems biology

## Abstract

**Background:**

Colorectal cancer arises from the accumulation of genetic mutations that induce dysfunction of intracellular signaling. However, the underlying mechanism of colorectal tumorigenesis driven by genetic mutations remains yet to be elucidated.

**Results:**

To investigate colorectal tumorigenesis at a system-level, we have reconstructed a large-scale Boolean network model of the human signaling network by integrating previous experimental results on canonical signaling pathways related to proliferation, metastasis, and apoptosis. Throughout an extensive simulation analysis of the attractor landscape of the signaling network model, we found that the attractor landscape changes its shape by expanding the basin of attractors for abnormal proliferation and metastasis along with the accumulation of driver mutations. A further hypothetical study shows that restoration of a normal phenotype might be possible by reversely controlling the attractor landscape. Interestingly, the targets of approved anti-cancer drugs were highly enriched in the identified molecular targets for the reverse control.

**Conclusions:**

Our results show that the dynamical analysis of a signaling network based on attractor landscape is useful in acquiring a system-level understanding of tumorigenesis and developing a new therapeutic strategy.

**Electronic supplementary material:**

The online version of this article (doi:10.1186/s12918-016-0341-9) contains supplementary material, which is available to authorized users.

## Background

Tumorigenesis cannot be explained by gene alterations themselves, but is rather becoming perceived as the resulting dysfunction of signaling pathways [1–3]. Signaling pathways carry external signals from the receptor to intracellular biological system, and the activation of signaling pathways is closely linked to the operation of specific biological processes. Therefore, malfunctioning of some crucial signaling pathway(s) due to genetic mutations can cause tumorigenesis [1, 4]. In particular, colorectal cancer, the most frequently occurring human cancer worldwide, is known to be caused by driver mutations such as tumor suppressor genes adenomatous poly-posis coli (APC), phosphatase and tensin homolog (PTEN), tumor protein p53 (TP53), and oncogene kirsten-ras (KRAS) [5–7]. In recent studies, the up-regulated Wnt signaling due to APC mutation or the growth signaling owing to KRAS mutation is considered as an important cause of tumorigenesis [8]. Moreover, the influence of such mutation has been observed affecting not only an individual signaling pathway but also the whole signaling flow in the signaling network in a complicated way due to crosstalks, feedback loops, etc. Therefore, in order to investigate the underlying mechanism of tumorigenesis from a signaling network perspective, we need to construct a large-scale human signal transduction network including various canonical signaling pathways and to develop a mathematical model for systematic analysis.

Mathematical modeling has been widely used to study the dynamics of a signaling pathway. In particular, continuous variable modeling based on a system of differential equations has often been used, but the estimation of kinetic parameter values limits its use for modeling a large-scale signaling network. To overcome such difficulty, we have employed in this study a Boolean network model based on integrated experimental evidence of signal transduction. In the Boolean network model, the state value of each node represents the activity of a signaling protein in a simplified way, ‘0’ for an inactive state and ‘1’ for an active state. The state of a signaling protein represented by discrete Boolean logic ultimately converges to an ‘attractor’ state after multiple state transitions. Here, an attractor is a mathematical concept representing a fixed stable state (point attractor) or regularly recurring states (cyclic attractor) adopted by a dynamic system [9–11]. Based on the concept of an attractor, the steady state characteristics of a biological network can be found from an attractor landscape where each point in the landscape represents a network state defined by a set of state values that contain the activity states of all the molecules in the network [10].

Colorectal tumorigenesis is known to be an evolutionary process which arises through sequential accumulation of somatic mutations, and the temporal order of genetic changes along tumorigenesis is crucial for tumor progression [6, 12]. Among driver mutations, the mutation of APC is the earliest genetic alteration in colorectal tumorigenesis and seems to be required for adenoma formation [13]. Activating KRAS mutation is an intermediate event of colorectal tumorigenesis and was observed in approximately 50 % of colorectal adenomas and carcinomas [14]. The mutated APC and KRAS genes have synergistic effect for clonal expansion in the nascent colorectal tumor. Relatively, TP53 mutation, observed in 75 % of colorectal carcinomas, is associated with the late progression of tumor rather than initiation [14, 15].

From the view of attractors, tumorigenesis, driven by sequential accumulation of somatic mutations, can be characterized by deformation of point or cyclic attractors of complex signal transduction networks. However, it has not been fully understood yet how network perturbations, such as somatic mutations, changes the attractor landscape during the development of cancer. In this study, to investigate the underlying mechanism of colorectal tumorigenesis at a system-level, we have reconstructed a large-scale human signaling network by integrating a vast amount of experimental evidence from literatures and previously published models, and then developed its Boolean logic model for dynamical analysis. Next, we conducted extensive analysis of the attractor landscape for colorectal tumorigenesis to reflect the sequential accumulation of driver mutations (APC, KRAS, PTEN and TP53). As a result, we found that the basin of a cancer progression attractor, characterized by abnormally regulated proliferation and metastasis, becomes larger along with the accumulation of driver mutations, which implies that the cellular state can prone to transit to a malignant state by the influence of driver mutations. In particular, KRAS and TP53 mutations, known as frequently observed mutations in patients of colorectal cancer, can significantly induce the change of a cellular state to a carcinoma state in concert with other mutations. Furthermore, based on the attractor landscape analysis of colorectal tumorigenesis, we performed a hypothetical investigation for reverse controlling to restore a normal phenotype from the cancerous state by regulating the activity status of specific target nodes in the signaling network. As a result, we could identify a set of minimal control nodes that can reshape the attractor landscape to drive all initial states of the attractor landscape into desired normal proliferative or quiescent phenotype attractors. Intriguingly, we found that approved drug-targets are significantly enriched in the set of identified control nodes. Our study provides new therapeutic insights and a way of discovering novel drug targets based on attractor landscape analysis and reverse control for the treatment of cancer which is known to be irreversible.

## Methods

### Construction of a Boolean network model of the human signaling network

To reconstruct a large-scale Boolean network model of the human signaling network, we have integrated previously published Boolean network models by Helikar et al., Fumia et al., Choi et al. and Kim et al., and then further added missing signaling components and interactions that are known to have crucial roles in signal transduction, from databases such as Kyoto Encyclopedia of Genes and Genomes (KEGG) and PID (Pathway Interaction Database) [10, 16–20]. The reconstructed human signaling network is composed of 197 nodes and 688 links. All the information of 688 links in the network is described in Additional file 1. In addition, for simulation analysis, we have constructed Boolean logic tables that describe the activity state of each node. The logic tables were constructed mainly based on the logic information of previously published Boolean models [10, 18–20]. For the newly added nodes and links, we performed an extensive literature survey to define the logical relationship of each signaling component based on experimental evidence. The descriptions of Boolean logic tables are included in Additional file 2 and the logic tables for each signaling component are summarized in Additional file 3. The Boolean simulation for attractor landscape analysis and identification of control nodes described in the following sections were all implemented using Matlab® 2014a and Python 2.7 in a Window Cluster system composed of 288 CPUs in parallel. The source codes for mathematical simulations were included in Additional file 4.

### Input–output relationship of the human signaling network

To investigate the biological properties of the human signaling network, we analyzed the input–output relationship of the network [19, 21]. In the Boolean simulation, the states of all nodes in the network were updated according to their assigned logical rules at each simulation step. The state values of each node were represented as ‘1’ or ‘0’, which means ‘ON’ or ‘OFF’, respectively. To compare the activities of the nodes in detail, we defined the steady-state activity of a node as the average activity over the last 100 time steps of the simulation over 1,000 time steps. For instance, if the activity of a node was observed as a cycle of ‘1010101010’, the steady-state activity of the node is determined to be 50 % ON. In the same manner, the input intensity was defined by the average ON of the external-input node during the simulation. We changed the input intensity to a range of 0-100 % ON with 1 % interval, and observed the steady-state activity of the node from different randomized initial conditions. We carried out additional simulations to investigate the influence of the last average steps by changing the steps to 30, 50 and 150 (see Figure S4 in Additional file 5). These results showed that input/output relationship of representative nodes was maintained regardless of an average step. It should be noted that, when we changed the input intensity of one input node, the activities of other input nodes were fixed to the optimal input settings, as adopted from Helikar et al. [19].

### Attractor landscape analysis of the human signaling network

To investigate the transition in the cellular state of the human signaling network during colorectal tumorigenesis, we performed the attractor landscape analysis of the human signaling network [10, 21, 22]. The attractor landscape is composed of attractors and their basins of attraction where an attractor is one of the steady-states of the network and the basin of an attractor represents a set of network states that converge to the attractor. For a given input condition, we obtained the attractor landscape of the human signaling network from randomly sampled 10,000 initial states until the state trajectory of an initial state reaches a point attractor with a fixed state or a cyclic attractor with periodically repeated states. It should be noted that our result was not significantly changed depending on the sampling size of the initial states (see Figure S1 in Additional file 5). We have reflected the genetic mutation events to our network by pinning the state of the mutated node as 1 or 0 when the mutation is gain-of-function or loss-of-function, respectively.

### Identification of control nodes and statistical analysis

To find out control nodes for reverse control, we adopted the algorithm for identifying the control kernel which is the minimal set of nodes that ensures all the initial states converge to a desired attractor [22]. Contrary to Kim et al., the aim of control in this study is not ensuring such convergence to a single attractor but ensuring convergence to attractors that are classified into groups characterized by a specific phenotype; quiescence or normal proliferation. In this respect, we have investigated a minimal set of control nodes that are required to be controlled to ensure that all initial states converge to the desired phenotypic attractors. The state values of the control nodes were fixed to either 0 or 1 during the simulation. Considering the large size of the constructed human signaling network, which computationally limits obtaining the whole attractor landscape of the network, 1,000 state transition trajectories were randomly sampled from different initial states to estimate the attractor landscape. We could identify control nodes by using the genetic algorithm (GA) which is a computational algorithm for optimization problems by artificially evolving chromosomes that contain all possible solutions. In this simulation, we adopted the fitness function described in Kim et al., and we used 100 initial chromosomes for GA [22]. The fitness function is as follows:

$$ Fitness=\left\{\begin{array}{c}\hfill {B}^3\times {\left(n-{\displaystyle {\sum}_{i=1}^n{X}_i}\right)}^2\times 2,\kern0.75em  if\kern0.5em B=1\hfill \\ {}\hfill {B}^3\times {\left(n-{\displaystyle {\sum}_{i=1}^n{X}_i}\right)}^2,\kern0.5em  otherwise\hfill \end{array}\right. $$

where *B* is the relative basin size of the desired phenotype, *n* is the number of nodes in the network, and *X*_*i*_ is 1 or 0 when node *i* is selected or not in a chromosome *X*, respectively. Then, the set of control nodes is determined by a node set in the chromosome with the highest fitness value (see Figure S3 in Additional file 5). Next, we retrieved the drug targets of the US Food and Drug Administration (FDA)-approved drugs from DrugBank database [23, 24], and conducted one-sided two-sample chi-squared tests to investigate the statistical significance between the control nodes and randomly selected nodes in the human signaling network.

## Results

### Boolean network model of the large-scale human signaling network

**Fig. 1:**
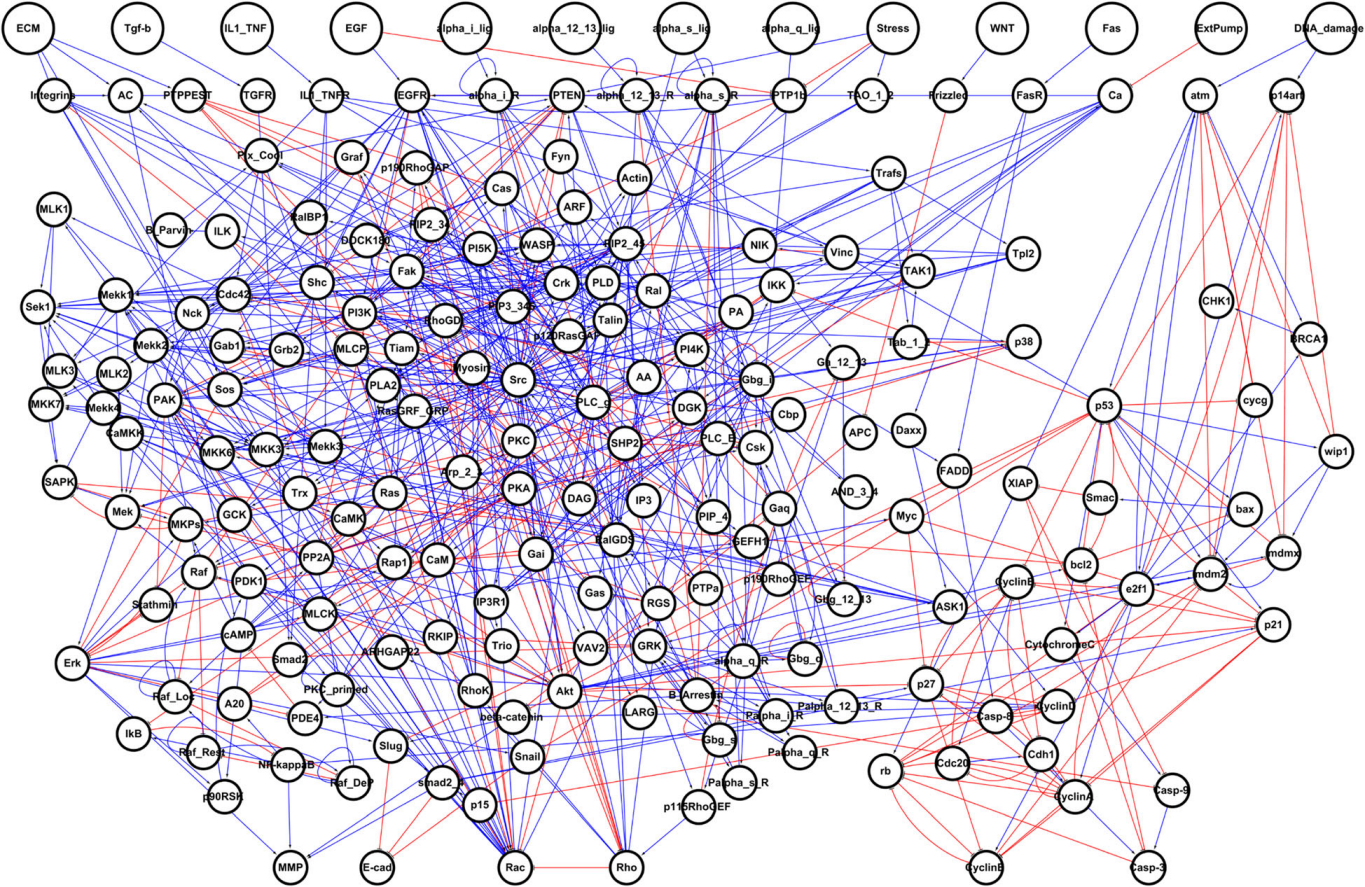
The human signaling network. The large-scale human signaling network consists of 197 nodes and 688 links; blue color, pointed arrows mean positive regulation links and red color, blunted arrows mean inhibitory regulation links. Among 197 nodes, there are 13 external-input nodes (see Additional file 1). The input nodes are represented by large circles

**Fig. 2:**
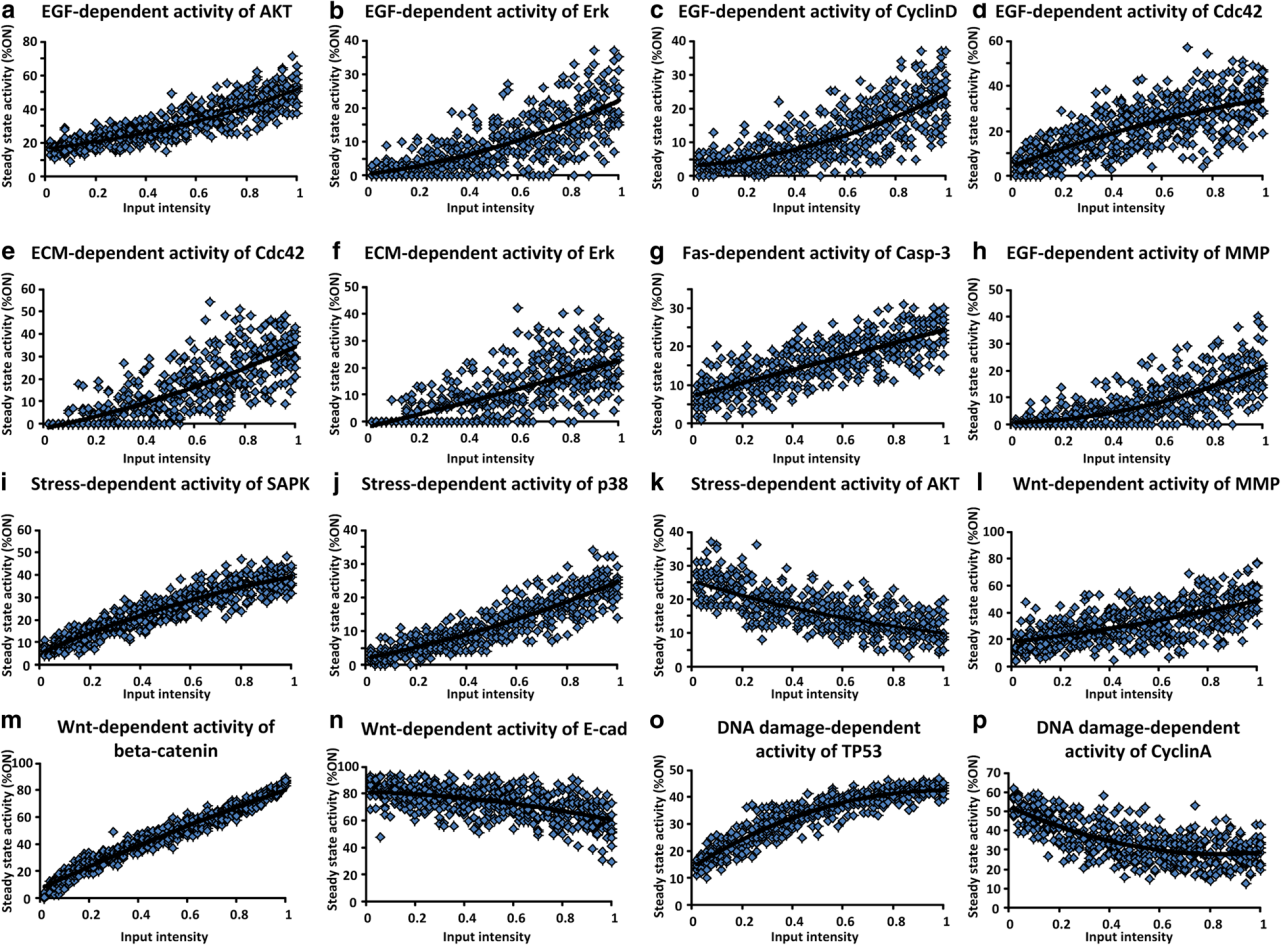
Qualitative input–output relationships in the Boolean model of human signaling network. **a** Positive relationship between EGF and AKT activation [62]. **b** Positive relationship between EGF and ERK activation [63]. **c** Positive relationship between EGF and CyclinD activation [34]. **d** Positive relationship between EGF and Cdc42 activation [64]. **e** Positive relationship between ECM and Cdc42 activation [65]. **f** Positive relationship between ECM and ERK activation [66]. **g** Positive relationship between Fas and Casp-3 activation [67]. **h** Activation of MMP by EGF [68]. **i-j** Stress-induced activation of SAPK and p38 [63, 69]. **k** Stress-induced inhibition of AKT [70]. **l-m** Wnt-induced activation of MMP and beta-catenin [71]. **n** Negative relationship between Wnt and E-cadherin [71]. **o** Positive relationship between DNA damage and TP53 activation [72]. **p** Negative relationship between DNA damage and CyclinA activation [73]. Note that the dose–response curves shown here are intended to demonstrate how the human signaling network model qualitatively reproduces the known input–output relationships over a wide range of inputs

To investigate the complex dynamics of intracellular signaling process, we have integrated all relevant information of key proteins and their interaction which are known to have a major role for biological processes from extensive manual curation of literatures and databases such as Kyoto Encyclopedia of Genes and Genomes (KEGG) and PID (Pathway Interaction Database) [16, 17]. In addition, we also retrieved previously published Boolean network models in Helikar et al., Fumia et al., Choi et al. and Kim et al., and integrated the information of network components and their mechanistic relations used in these network models (See Methods for details) [10, 18–20]. The reconstructed human signaling network consists of 197 nodes and 688 directed links, and contains thirteen external-input nodes; extracellular matrix (ECM), epidermal growth factor (EGF), interleukin tumor necrosis factor (IL1_TNF), transforming growth factor-beta (TGF-beta), G protein-coupled receptors (GPCR) ligands (alpha_i_lig, alpha_12_13_lig, alpha_s_lig, alpha_q_lig), Stress, Wnt, Fas, calcuim pump (Extpump) and DNA damage (Fig. 1 and Additional file 1). External-input nodes in the human signaling network receive signals propagated by different external stimuli and activate downstream signaling pathways. The reconstructed human signaling network involves various signaling pathways, including mitogen activated protein kinase (MAPK), phosphoinositide 3-kinase (PI3K)/AKT, Wnt, TGF-beta, TP53, calcium, DNA damage-related ATR/ATM, Rho GTPases, tumor necrosis factor-alpha (TNF alpha), and p38/JNK pathways, which have critical roles in determining cellular functions. The main function of each signaling pathway is well-known from previous studies [25–27]. For instance, MAPK, PI3K/AKT, TGF-beta, and TNF-alpha signaling pathways perform roles associated with the growth or death of cells, and Wnt and Rho GTPases pathways regulate cellular adhesion and migration of cells. In addition, ATR/ATM and p38/JNK signaling pathways play key roles in determining the response of cells to stress and DNA damage signals from the extracellular environment. The reconstructed human signaling network is adequate to investigate the distinct cellular phenotypes and complex biological processes because it includes core signaling pathways with various crosstalks and feedback loops. Furthermore, in order to investigate the dynamics of the human signaling network, a discrete Boolean network model of the network was established based on the mechanistic relations of each network component. The logic tables which determine the activity state of each network component were constructed based on relevant literatures and previously published Boolean network models (see Methods for details). In addition, qualitative simulations were performed by varying different inputs from 0 % to 100 % to examine the model’s ability to reproduce biological properties of the real human signaling network. The results indicate that the human signaling network model successfully reflects the qualitative features of the known biological activities of the network components (Fig. 2).

### Colorectal tumorigenesis upon the attractor landscape

Complex signaling networks exhibit dynamical properties, leading to a multitude of cellular behaviors or phenotypes, each representing an attractor state. An attractor is defined as a stable state of a dynamic system, in which a wide variety of initial states move toward a specific state of the system that tends to converge over time. In particular, for biological networks, the concept of an attractor can be used to map the dynamic behavior of the network into an attractor landscape. A set of all initial states that converge to a given attractor is defined as the basin of the attractor.

In the attractor landscape analysis, the attractors determined by the activation patterns of network components are indicators of distinct cell phenotypes, and the cell phenotypes can be defined by the part of the representative nodes instead of whole nodes in the network. In this study, we defined a few basic cell phenotypes considering the effects of mutations. The basic cell phenotypes are apoptotic, metastatic, proliferative and quiescent phenotypes. The apoptotic phenotype is characterized by active cysteine-aspartic proteases (caspases), and the metastatic phenotype is determined by inactive E-cadherin, active matrix metalloproteinases (MMP) and Rho oscillation. Wnt and Rho GTPases signaling stimulations induced by receptor tyrosine kinases (RTK) activation have a crucial role in tumor progression toward a highly invasive malignant phenotype [28–30]. Among the components of both signaling pathways, loss of E-cadherin with active MMP-mediated cell adhesion and the occurrence of Rho oscillation-mediated push‐pull mechanism for cell migration are a prerequisite condition for the subsequent formation of tumor metastases [18, 31–33]. The proliferative phenotype is decided when cyclins are activated along the cell cycle in the correct sequence (CyclinD → CyclinE → CyclinA → CyclinB → CyclinD), and the quiescent phenotype is determined when both proliferative and metastatic phenotypes are not given. In particular, the proliferative attractor where the node CyclinD remains active along the cyclic transition is defined as an abnormal proliferative phenotype. CyclinD is a well-known human oncogene and activator of cell cycle progression. The overexpression of CyclinD is known to cause a number of potentially oncogenic responses and many different types of cancer commonly have CyclinD overexpression rates of 15 ~ 40 % [34]. Each attractor can be categorized according to three criteria based on cell phenotype classifications: (1) whether normal regulation of a proliferative activity is possible, (2) whether the proliferative phenotype is shown, and (3) whether the metastatic phenotype is shown. The attractors were classified into eight types according to the attractor classification (See Figure S2 in Additional file 5). Among eight types of attractor, two attractors were designated as normal proliferation and cancer progression attractors. The normal proliferation attractor was defined that the normal regulated proliferation is operated without any metastatic behavior. The cancer progression attractor was defined as having the abnormal proliferation operated with metastatic activity in ON state.

In the case of colorectal cancer, tumorigenesis has been known as a multistep process, which can be caused by a combination of genetic mutations [6, 14]. These genetic mutations induce inappropriate activation or inactivation of specific signaling or genes, which drives the transition of a cellular state from adenoma to carcinoma during tumorigenesis. Among various genetic mutations, the inactivation of the tumor suppressor genes, APC, PTEN, and TP53 and the activation of the oncogene, KRAS are considered to be critical factors in colorectal tumorigenesis [6, 35–37]. In addition, the order of these genetic mutations is well-studied (i.e., APC deletion followed by KRAS over-activation, then PTEN deletion, and finally TP53 deletion) [20, 38]. Based on these, we have investigated the effect of sequential genetic mutations for colorectal tumorigenesis by simulating the corresponding node perturbations in the attractor landscape. In this analysis, we set up the external input condition as a normal growth state, which includes EGF, ECM and Wnt external inputs. Each external signal is associated with cell growth, cytoskeletal regulation, and cell migration, respectively. Such an external input condition was provided in the same way for each attractor landscape analysis of the Boolean network model.

**Fig. 3:**
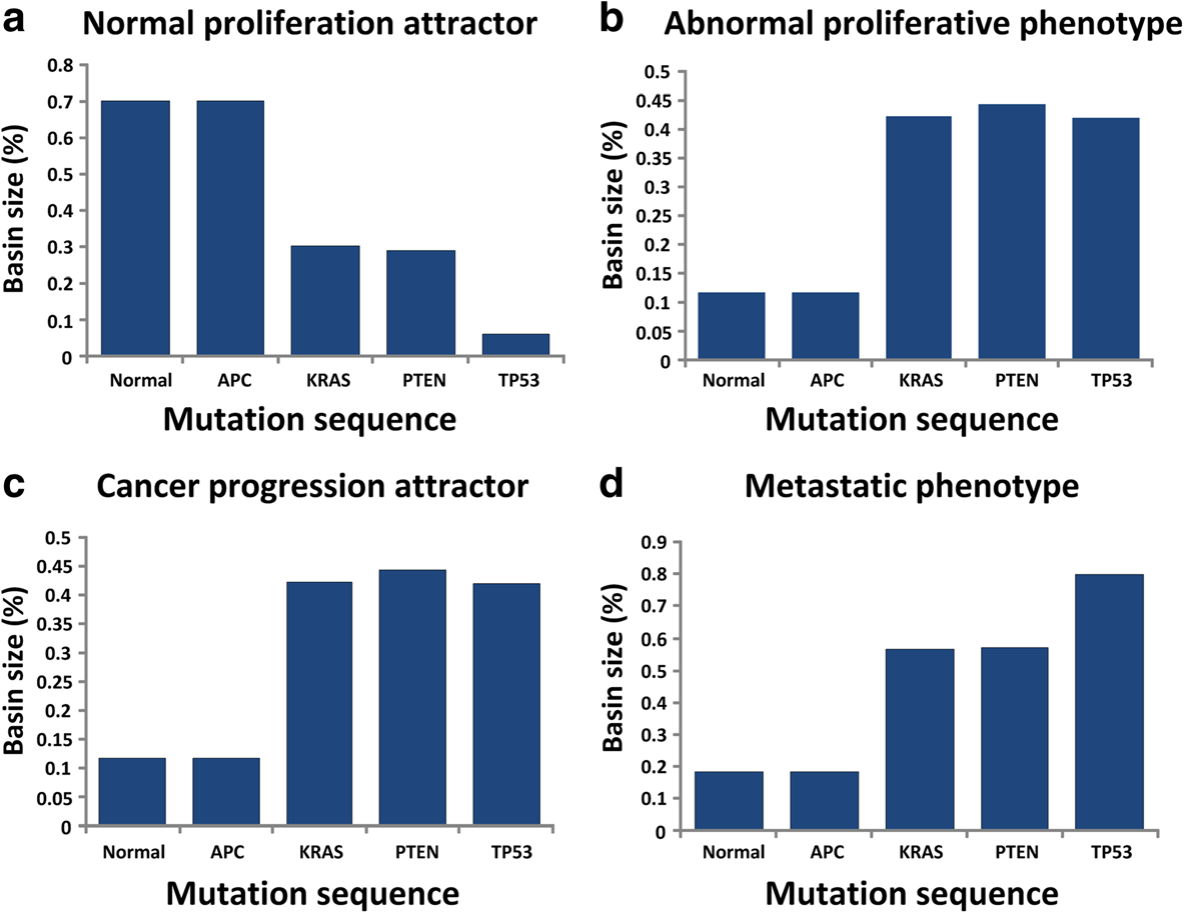
The change of attractor basin along with the sequential accumulation of driver mutations during colorectal tumorigenesis. **a** The basin size of a normal proliferation attractor. The basin size of a normal proliferation attractor was decreased by KRAS and TP53 mutations. **b** The basin size of an abnormal proliferative phenotype attractor. The basin size of an abnormal proliferative phenotype attractor was significantly increased by KRAS mutation. **c** The basin size of a cancer progression attractor. The basin size of a cancer progression attractor was increased by KRAS mutation. **d** The basin size of a metastatic phenotype attractor. The basin size of a metastatic phenotype attractor shows an increasing tendency by sequential accumulation of driver mutations. In particular, the mutations of KRAS and TP53 dramatically increased the size of a metastatic phenotype attractor

The first mutation introduced in the network was APC deletion. In the case of APC mutation, there was no significant change in the attractor landscape and the basin size of each attractor (Fig. 3). Next, along with the sequence of driver mutations, constitutive activation of KRAS was introduced in the network model. In this case, the basin size of a normal proliferation attractor was significantly decreased from 70.4 % to 30.4 % compared to that of the network without any genetic mutation and the basin size of cancer progression, abnormal proliferative phenotype and metastatic phenotype attractors were dramatically increased from 11.8 % to 42.2 %, from 11.8 % to 43.4 % and from 18.4 % to 56.9 %, respectively (Fig. 3b, c, d). Loss of function of APC tumor suppressor gene is thought to initiate neoplastic growth, and activating mutations of KRAS oncogene are commonly associated with tumor progression from a benign adenoma to a dysplastic adenocarcinoma [14, 39]. In our case, since KRAS mutation affects the key downstream regulators, ERK and MEK signaling components involved in the MAPK signaling pathway, suppression of normal proliferation and enlargement of the abnormal proliferation ratio were observed as an indicator of the benign adenocarcinoma during colorectal tumorigenesis. In addition, activated KRAS is known to trigger tyrosine phosphorylation of beta-catenin, leading to its release from E-cadherin at the adherence junction and the increase of Wnt signal transduction to the nucleus [40, 41]. Owing to the underlying interaction with beta-catenin, we found that KRAS mutation increases the basin size of a metastatic phenotype attractor. This result indicates that KRAS mutation can accelerate the metastatic phenomena during colorectal tumorigenesis. According to the sequence of driver mutations, PTEN was deleted. The ratios of abnormal proliferative phenotype and cancer progression attractors were slightly increased from 43.4 % to 45.7 % and from 42.2 % to 44.5 %, respectively, and the basin size of a normal proliferation attractor was decreased from 30.4 % to 28.9 %, but no remarkable change was observed (Fig. 3a, b, c). The last mutation in the sequence of driver mutations was TP53 deletion. TP53 mutation is considered a relatively late event in the development of colorectal cancer. In our simulation result, the basin size of a normal proliferation attractor was dramatically decreased from 28.9 % to 5.9 % and the basin size for a metastatic phenotype was increased from 57 % to 80 % (Fig. 3a, d). This result indicates that TP53 can be an important determinant of cancer progression from adenoma to a malignant and metastatic tumor state.

In summary, as shown in Fig. 3, our simulation results show that sequential driver mutations for the colorectal tumorigenesis contributes to increasing both abnormal proliferation and metastasis. In the attractor landscape, 43.4 % of the initial states converged to the abnormal proliferative phenotype while only 11 % converged to that in the absence of driver mutations, and the basin for the metastatic phenotype increased from 18.4 % to 80 % by the sequential genetic mutations. In particular, among the driver mutations, KRAS and TP53 mutations, known as frequently observed mutations in colorectal cancer, induced the change of a cellular state from benign adenoma to carcinoma in concert with other mutations during colorectal tumorigenesis. Together, the attractor landscape analysis of the Boolean network model elucidates the system-level mechanism underlying colorectal tumorigenesis driven by driver mutations.

### Reverse control of colorectal tumorigenesis

Dysfunction of intracellular signaling is crucial in tumorigenesis [4, 42]. In particular, colorectal tumorigenesis is known to arise by sequential accumulation of somatic mutations. These sequential mutations cause rewiring of the molecular interaction network, which means that mutated cells would have different network dynamics. So, the genetic mutations that cause the change of network dynamics allow normal cells to transform into cancer cells.

In this study, we have investigated how cancer cells can be treated by controlling the molecular network dynamics. The complex signaling network should have a certain degree of functional redundancy to keep tolerance of its biological function to the failure of specific nodes or links. With regard to the functional redundancy, distinct signaling components in an alternative signaling pathway can compensate for the failure of components to sustain the activity of key downstream processes [43]. Considering such functional redundancy of the signaling network, we performed a hypothetical investigation for reverse controlling to restore a normal phenotype from the cancerous state by regulating the activity status of specific target nodes in the signaling network. To identify a set of minimal control nodes for the reverse control, we have employed genetic algorithm which is a computational optimization algorithm widely used to solve complex problems (see Methods for details) [22].

To identify control nodes for cancer reversion, we considered the following control objectives: achieving quiescent or normal proliferative phenotype. Each of these objectives might result in different therapeutic strategies for the treatment of cancerous cells [44]. The first control strategy for changing the cancerous phenotype into a quiescent phenotype can be used to suppress any further progression of cancer cells into malignant types by blocking the signaling for proliferation and metastasis in response to external signals such as EGF or Wnt. On the other hand, the second control strategy, changing the cancerous phenotype into a normal proliferative phenotype can be used for restoring the original phenotype of normal cells to recover the normal cellular functioning by making use of the functional redundancy of the complex signaling network. We have investigated such reverse control strategies and identified a set of minimal control nodes upon each deformed attractor landscape along with the cancer progression stage. The cancer progression stage is characterized by the accumulation level of driver mutations.

**Fig. 4:**
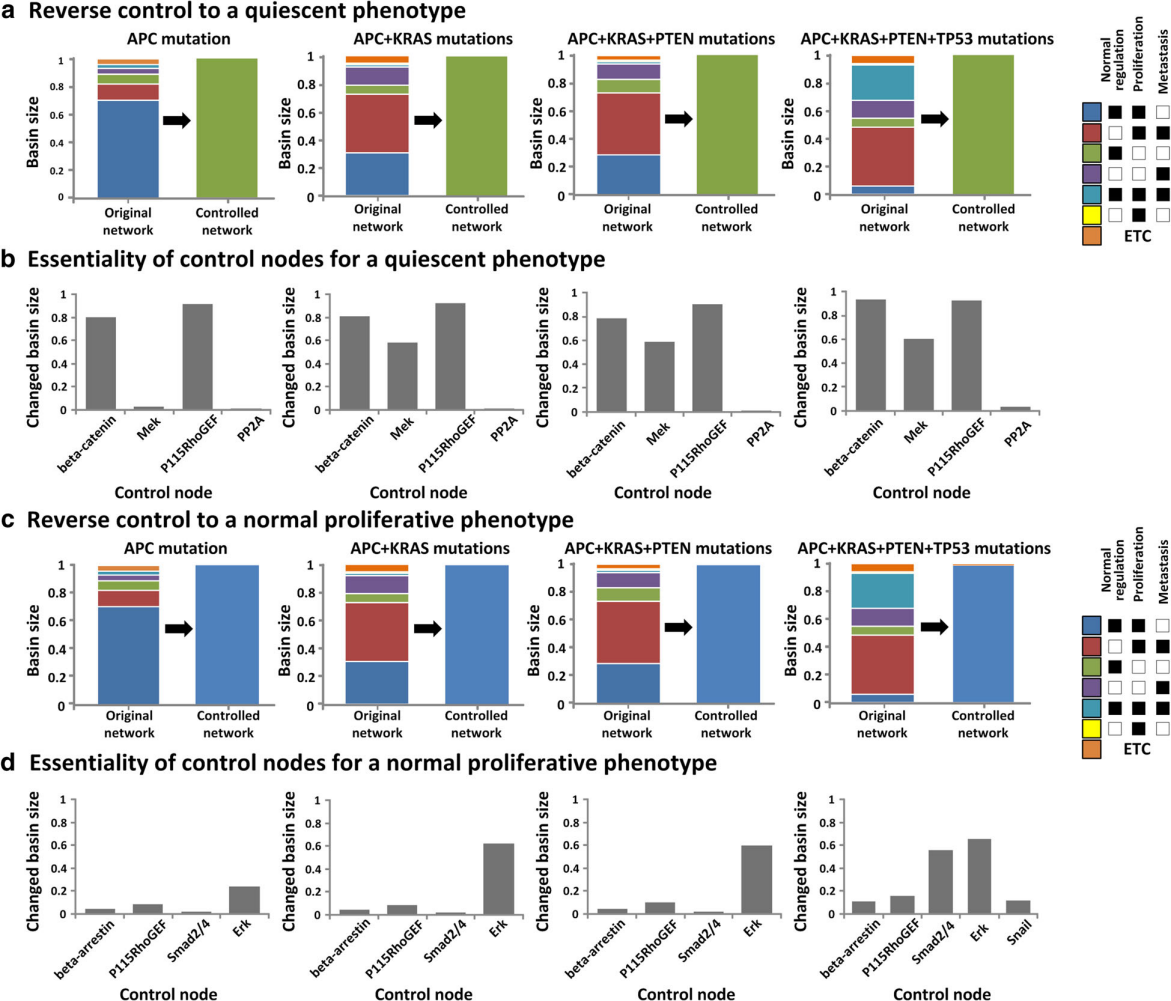
Distribution of the basin size for the phenotype attractors in the attractor landscape before and after performing reverse control and the essentiality of control nodes for each accumulation stage of driver mutations. **a** and **c** The results of reverse control for transforming the cellular state to a quiescent or normal proliferative phenotype. **a** Reverse control to a quiescent phenotype (*green color*). **c** Reverse control to a normal proliferative phenotype (*blue color*). We should reshape the attractor landscape at each accumulation stage of driver mutations to make all initial states of attractor landscape converge to the attractor of a quiescent or a normal proliferative phenotype by regulating the activity of control nodes. In the right panel, colored boxes represent the classified attractors that indicate different cellular phenotypes. More details about the attractor classification are described in the main text. **b** and **d** Essentiality of control nodes identified in the reverse control. **b** Essentiality of control nodes for a quiescent phenotype. **d** Essentiality of control nodes for a normal proliferative phenotype. The essentiality indicates the capability of each control node to change the original attractor landscape into the desired one

First, we have investigated the reverse control that can change the cellular phenotype to a quiescent phenotype for each cancer progression stage (Fig. 4a). As a result, we have identified a minimal set of control nodes including beta-catenin, MEK, Rho guanine nucleotide exchange factor-1 (P115RhoGEF) and protein phosphatase 2A (PP2A), that are essential for recovering a quiescent phenotype regardless of the cancer progression stage (Table 1). In particular, the inhibition of beta-catenin, P115RhoGEF and MEK with the activation of PP2A makes all initial states of the attractor landscape converge to a quiescent phenotype attractor. In addition, we found that the identified nodes play a critical role in changing the cellular phenotype by blocking alternative signal propagation through distinct signaling pathways. Beta-catenin has an important role for signal transduction as a component of the Wnt signaling pathway. Beta-catenin is activated by Wnt signal, and activates the downstream key genes associated with cell proliferation and metastasis, such as CyclinD, MMP and Slug [45]. Therefore, inhibition of beta-catenin can reduce abnormal proliferation and metastasis induced during colorectal tumorigenesis. In addition, P115RhoGEF, the regulator of G-protein signaling domain, transmits cell migration and adhesion signals from GPCR, and leads to the activation of Rho [46–48]. The down-regulation of P115RhoGEF inhibits the activity of Rho, which is critical for reducing cell migration. Together, we can successfully reduce the metastatic phenotype involved in cell migration and invasion of cancer cells by controlling the activities of beta-catenin and P115RhoGEF. Next, the inhibition of MEK and activation of PP2A have a critical role to reduce the abnormal proliferation of cancer cells. MEK is a signaling component involved in the MAPK signaling pathway which transmits a cell growth signal through the protein kinase cascade (known as RAS → RAF → MEK → ERK) [49]. So, the inhibition of MEK strongly blocks the growth signal transduction. In addition, PP2A, which is known as a tumor suppressor, induces the change in the phosphorylation status of AKT and ERK [50]. In particular, the up-regulated PP2A blocks the cell growth signal that is transmitted through the PI3K signaling pathway by inhibiting the activation of AKT. Therefore, the combined blockage of the MAPK and PI3K signaling pathways through down-regulation of MEK and PP2A activities can significantly decrease the abnormal proliferation during cancer development [51].

**Table 1:** The minimal sets of control nodes for the reverse control

APC mutation	APC + KRAS mutations	APC + KRAS + PTEN mutations	APC + KRAS + PTEN + TP53 mutations	Control direction
Control nodes for a quiescent phenotype				
beta-catenin	beta-catenin	beta-catenin	beta-catenin	
P115RhoGEF	P115RhoGEF	P115RhoGEF	P115RhoGEF	
MEK	MEK	MEK	MEK	
PP2A	PP2A	PP2A	PP2A	
Control nodes for a normal proliferative phenotype				
beta-arrestin	beta-arrestin	beta-arrestin	beta-arrestin	
Erk	Erk	Erk	Erk	
P115RhoGEF	P115RhoGEF	P115RhoGEF	P115RhoGEF	
Smad2/4	Smad2/4	Smad2/4	Smad2/4	
			Snail	

Next, we have investigated the reverse control to a normal proliferative phenotype (Fig. 4c). The attractor of a normal proliferative phenotype represents a cellular state that shows normal proliferation without any metastatic behavior, which should be a dominant attractor of normal cells (i.e. networks without any mutation) and actually occupies 60 % of the attractor landscape. As a result of restoring the network state in each cancer progression stage, we have identified different sets of a minimal number of control nodes including beta-arrestin, P115RhoGEF, Smad2/4, ERK and Snail (Table 1). From adenoma (a network carrying a mutation in APC) to the late adenocarcinoma stage (a network with mutations in APC, KRAS, and PTEN), we found that it is possible to drive all initial states of the attractor landscape into the attractor of a normal proliferative phenotype by changing the status of four target nodes, beta-arrestin, P115RhoGEF, Smad2/4, and ERK. However, in the carcinoma stage (a network with mutations in APC, KRAS, PTEN, and TP53), Snail is additionally required as a control node, and we can drive in this case the entire 98 % of the initial states into a desired attractor by regulating the activities of five control nodes.

In our network model, we found that the identified control nodes modulate the core signaling to restore a normal proliferative phenotype of cells in a very complicated way. For instance, P115RhoGEF, in addition to the aforementioned role, serves to reduce the metastatic phenotype by regulating the activity of Rho. And, the down-regulation of ERK, involved in the MAPK signaling pathway, decreases the abnormal proliferation of cancer cells. Beta-arrestin is activated by GPCRs and activates the MAPK and PI3K/AKT signaling pathways by Src activation [52, 53]. Beta-arrestin binds to several members of Src and engages them to activate GPCRs as well as the downstream signaling pathways [54]. The inhibition of beta-arrestin serves to block the growth-related signal transduction during cancer development. The activation of Smad2/4 causes the invasion and metastasis of cancer cells by inducing the activity change of E-cadherin [55]. Therefore, the down-regulation of Smad2/4 promotes to facilitate the restoration of a normal proliferative phenotype by reducing the metastatic behavior of tumor cells. The last control node, Snail is additionally selected as a control node in the carcinoma stage of colorectal tumorigenesis. Snail has a role to suppress the activity of E-cadherin, and interacts with MMP in tumor metastasis [56]. A recent study showed that TP53 modulates Snail-induced tumor metastasis by Snail degradation [57]. This implicates that, in the carcinoma stage, the inhibition of Snail is additionally required to reduce the metastatic phenotype increased by the loss of function of TP53. Together, our study suggests that the inhibitory control of beta-arrestin, P115RhoGEF, Smad2/4, ERK and Snail might be able to restore the normal proliferative phenotype from cancerous state in colorectal tumorigenesis.

### Essentiality of control nodes and the enrichment analysis of approved drug-targets

To investigate the effectiveness of each control node, we measured the essentiality of control nodes as the ratio of a desired attractor basin after we controlled the activity of each control node. The essentiality of each control node is represented by a value between zero and one, on a scale of increasing the influence over the reverse control. In this study, we found that the identified control nodes for reversely controlling the cellular state into a normal proliferative or quiescent state were almost same regardless of the stage of cancer development, whereas the essentiality of each control node was quite different along with the accumulation level of driver mutations.

First, we calculated the essentiality of four control nodes, beta-catenin, MEK, P115RhoGEF, and PP2A, which were identified for the reverse control to a quiescent phenotype (Fig. 4b). In the benign adenoma stage, the essentiality of beta-catenin and P115RhoGEF was observed to be as high as 0.804 and 0.919 whereas that of MEK and PP2A was observed to be as relatively low as 0.024 and 0.01, respectively. However, in the adenocarcinoma stage in which the KRAS mutation was added, the essentiality of MEK increased dramatically from 0.024 to 0.58. This result indicates that the activity control of MEK became more important than in the previous stage to suppress abnormal proliferation elevated by the over-expression of KRAS. In the late adenocarcinoma stage, there was no significant change in the essentiality of each node. Lastly, in the carcinoma stage that was preceded by the mutation of TP53, the essentiality of all control nodes (i.e., beta-catenin, P115RhoGEF, MEK, and PP2A) was significantly increased (Fig. 4b). The occurrence of TP53 mutation increased the basin of metastatic and abnormal proliferative phenotype attractors. Therefore, we need stronger regulation of control nodes than the previous stages of colorectal tumorigenesis.

Next, we have investigated the essentiality of each target node for a normal proliferative phenotype (Fig. 4d). In the benign adenoma stage, the essentiality of four control nodes, beta-arrestin, P115RhoGEF, Smad2/4, and ERK were observed as 0.039, 0.085, 0.017, and 0.239, respectively. In the normal state, the attractor of normal proliferative phenotype was represented as a primary attractor which occupied 60 % of the attractor landscape of the original network. Therefore, because the remaining proportion of an attractor landscape was relative small, the essentiality values of target nodes show relatively low values compared to those of other control nodes. In the adenocarcinoma stage, we found that the essentiality of ERK was significantly increased since it reduces the abnormal proliferative phenotype elevated by KRAS over-expression. Finally, in the carcinoma stage, the essentiality of Smad2/4 and Snail was vastly increased from 0.017 to 0.561 and from 0 to 0.116, respectively, in order to reduce the metastatic behavior of malignant cells that was increased by the accumulation of TP53 mutation.

**Fig. 5:**
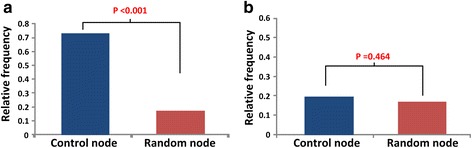
The enrichment of approved drug-targets in the identified control nodes and the random nodes of the human signaling network. **a** The enrichment of approved drug-targets in the control nodes for a quiescent phenotype and the random nodes. **b** The enrichment of approved drug-targets in the control nodes for a normal proliferative phenotype and the random nodes. Control nodes are listed in Table 1

For further analysis, we compared the enrichment of approved drug-targets in the control nodes to that of randomly selected nodes in the human signaling network. Changing the network state into a desired state by controlling the activity of specific nodes is associated with controlling the dynamics of a cellular system. This control strategy suggests that the control nodes might be associated with drug targets. Intriguingly, we found that the approved drug-targets are significantly enriched in the control nodes for a quiescent phenotype compared to randomly selected nodes (p-value < 0.001) (Fig. 5a). In contrast, the enrichment of approved drug-targets in the control nodes for a normal proliferative phenotype showed a relatively similar frequency compared to randomly selected nodes (p-value = 0.464) (Fig. 5b). Taken together, our study provides a new therapeutically beneficial strategy to discover novel drug targets for the cancer treatment.

## Discussion

Cancer is a well-studied complex disease and extensive efforts have been made to explore tumorigenesis during the past few decades [3]. Although it is known that a wide variety of genetic changes contribute to the abnormal signaling in tumorigenesis, it still remains as a challenge to obtain a global view of how they affect the signaling alterations to develop cancer in the entire signaling network [4]. A cellular signaling network consists of various signal pathways interlinked by complex regulatory relationships such as feedbacks and crosstalks. This complexity leads to a fundamental limitation in using only conventional biological experimental techniques to understand the cellular behavior. To overcome such a limitation, mathematical modeling and computer simulations were employed in this study to explore the hidden mechanism of complex signal transduction systems.

Cancer reversion, reversing tumorigenesis which is known to be an irreversible process, was theoretically suggested somewhile ago and there have been several experimental studies testing this concept. For instance, the cancerous crypt with suppressed APC showed the tumor regression and re-establishment of a normal crypt was observed after restoring the activation of APC [58]. Cancerous cells from a leukemia patient were observed being transformed to cells like normal macrophages by myeloid reprogramming [59]. Reversal of the oncogene’s and tumor suppressor’s status was suggested to cause the reduced tumorigenicity by the direct reprogramming of cancer cells [60]. Such reverse control of the cancer can be an alternative treatment for cancer patients. Currently, the treatment of cancer mainly focuses on the strategy for killing tumor cells. This approach is limited by the resistance of the heterogeneous cancer population because only sensitive cells would be killed whereas resistant cancer cells can survive and eventually overwhelm the whole population after repeated rounds of treatment. The cancer reversion strategy can overcome such limitation because the reversed cells can still survive and compete with other heterogeneous cells in the tumor population [61]. However, there have been very few studies in this direction. In our study, based on the attractor landscape analysis, we suggested a novel strategy for cancer reversion that identifies control nodes by which the cancerous cell might lose its malignancy and finally become a quiescent or normal phenotype. Our results showed that approved drug-targets are highly enriched in the control nodes for a quiescent phenotype since previous studies of a drug target excavation for cancer treatment mainly focused on preventing growth or inducing apoptosis of cancer cells, which is a similar strategy to change the cellular state into a quiescent state. In contrast, the enrichment of drug targets in the control nodes for a normal proliferative phenotype was not significantly different from that of randomly selected nodes, since the strategy of changing the cancerous state into a normal state has not been thoroughly studied yet. This result indicates that the control nodes for a normal proliferative phenotype were not conventionally considered as drug targets, so our results should be validated by experiments in future studies.

## Conclusions

To investigate the underlying mechanism of colorectal tumorigenesis at a system-level, we have reconstructed a large-scale human signaling network by integrating all relevant information of canonical signaling pathways related to proliferation, metastasis, and apoptosis from extensive survey of literatures and databases. Moreover, we developed a discrete Boolean network model, and verified that the Boolean dynamics of a reconstructed network can reproduce relevant features of well-known input–output relationships of signaling network components. This model was then used for a system-level investigation of colorectal tumorigenesis based on the concept of an attractor landscape. Our attractor landscape analysis of colorectal tumorigenesis not only elucidated the progression mechanism driven by driver mutations, but also showed that this analysis can serve as a framework to identify a new drug target in the complex signaling network. From systems analysis, we found that the stage-wise progression of colorectal tumorigenesis can be explained by sequential accumulation of four driver mutations: APC, KRAS, PTEN, and TP53. In particular, we found that KRAS and TP53 mutations dramatically increased the basin of abnormal proliferation and metastasis attractors, respectively. Moreover, from a hypothetical investigation of cancer reversion, we identified a minimal set of control nodes to alter the cancerous phenotype into a quiescent or normal proliferative phenotype for each stage of cancer progression. It is remarkable that approved drug-targets were highly enriched in the identified control nodes for the reverse control. Our study provides a new system-level understanding of colorectal tumorigenesis and provides a promising new way of discovering a novel drug target for the cancer treatment.

## Additional files

Additional file 1: The 688 links comprising the human signaling network. (XLS 111 kb)
Additional file 2: The Boolean logic descriptions of nodes in the human signaling network. (XLS 3607 kb)
Additional file 3: The Boolean logic tables of nodes in the human signaling network. Each text file contains a logic table for individual signaling component of the human signaling network. The file name of each text file denotes a target molecule. In the files, each column except the last one displays the name of the source nodes and all possible activity conditions. The last column describes the activity state of the target nodes for each regulating condition of the source nodes. For the 197 nodes of the human signalling network, the 184 text files (except 13 input nodes) are included in Additional file 3. (ZIP 102 kb)
Additional file 4: The Matlab and Python source codes for mathematical simulations. (ZIP 1070 kb)
Additional file 5: Supporting Figures and Tables (contains 4 figures and 5 tables). (PDF 634 kb)

## Abbreviations

APC: Adenomatous poly-posis coli
caspases: Cysteine-aspartic proteases
ECM: Extracellular matrix
EGF: Epidermal growth factor
Extpump: Calcuim pump
FDA: US Food and Drug Administration
GA: Genetic algorithm
GPCR: G protein-coupled receptors
IL1_TNF: Interleukin tumor necrosis factor
KEGG: Kyoto Encyclopedia of Genes and Genomes
KRAS: Kirsten-ras
MAPK: Mitogen activated protein kinase
MMP: Matrix metalloproteinases
P115RhoGEF: Rho guanine nucleotide exchange factor-1
PI3K: Phosphoinositide 3-kinase
PID: Pathway Interaction Database
PP2A: Protein phosphatase 2A
PTEN: Tensin homolog
RTK: Receptor tyrosine kinases
TGF-beta: Transforming growth factor-beta
TNF alpha: Tumor necrosis factor-alpha
TP53: Tumor protein p53

## References

[1] Kolch W, Halasz M, Granovskaya M, Kholodenko BN (2015). The dynamic control of signal transduction networks in cancer cells. Nat Rev Cancer.

[2] Guney E, Menche J, Vidal M, Barabasi AL (2016). Network-based in silico drug efficacy screening. Nat Commun.

[3] Hanahan D, Weinberg RA (2000). The hallmarks of cancer. Cell.

[4] Cui Q, Ma Y, Jaramillo M, Bari H, Awan A, Yang S, Zhang S, Liu L, Lu M, O’Connor-McCourt M (2007). A map of human cancer signaling. Mol Syst Biol.

[5] Armaghany T, Wilson JD, Chu Q, Mills G (2012). Genetic alterations in colorectal cancer. Gastrointest Cancer Res.

[6] Smith G, Carey FA, Beattie J, Wilkie MJ, Lightfoot TJ, Coxhead J, Garner RC, Steele RJ, Wolf CR (2002). Mutations in APC, Kirsten-ras, and p53--alternative genetic pathways to colorectal cancer. Proc Natl Acad Sci U S A.

[7] Cully M, You H, Levine AJ, Mak TW (2006). Beyond PTEN mutations: the PI3K pathway as an integrator of multiple inputs during tumorigenesis. Nat Rev Cancer.

[8] Janssen KP, Alberici P, Fsihi H, Gaspar C, Breukel C, Franken P, Rosty C, Abal M, El Marjou F, Smits R (2006). APC and oncogenic KRAS are synergistic in enhancing Wnt signaling in intestinal tumor formation and progression. Gastroenterology.

[9] Huang S, Eichler G, Bar-Yam Y, Ingber DE (2005). Cell fates as high-dimensional attractor states of a complex gene regulatory network. Phys Rev Lett.

[10] Choi M, Shi J, Jung SH, Chen X, Cho KH (2012). Attractor landscape analysis reveals feedback loops in the p53 network that control the cellular response to DNA damage. Sci Signal.

[11] Ding S, Wang W (2011). Recipes and mechanisms of cellular reprogramming: a case study on budding yeast Saccharomyces cerevisiae. BMC Syst Biol.

[12] Fodde R, Smits R, Clevers H (2001). APC, signal transduction and genetic instability in colorectal cancer. Nat Rev Cancer.

[13] Powell SM, Zilz N, Beazer-Barclay Y, Bryan TM, Hamilton SR, Thibodeau SN, Vogelstein B, Kinzler KW (1992). APC mutations occur early during colorectal tumorigenesis. Nature.

[14] Vogelstein B, Fearon ER, Hamilton SR, Kern SE, Preisinger AC, Leppert M, Nakamura Y, White R, Smits AM, Bos JL (1988). Genetic alterations during colorectal-tumor development. N Engl J Med.

[15] Rodrigues NR, Rowan A, Smith ME, Kerr IB, Bodmer WF, Gannon JV, Lane DP (1990). p53 mutations in colorectal cancer. Proc Natl Acad Sci U S A.

[16] Kanehisa M, Goto S (2000). KEGG: kyoto encyclopedia of genes and genomes. Nucleic Acids Res.

[17] Schaefer CF, Anthony K, Krupa S, Buchoff J, Day M, Hannay T, Buetow KH (2009). PID: the Pathway Interaction Database. Nucleic Acids Res.

[18] Kim TH, Monsefi N, Song JH, von Kriegsheim A, Vandamme D, Pertz O, Kholodenko BN, Kolch W, Cho KH (2015). Network-based identification of feedback modules that control RhoA activity and cell migration. J Mol Cell Biol.

[19] Helikar T, Konvalina J, Heidel J, Rogers JA (2008). Emergent decision-making in biological signal transduction networks. Proc Natl Acad Sci U S A.

[20] Fumia HF, Martins ML (2013). Boolean network model for cancer pathways: predicting carcinogenesis and targeted therapy outcomes. PLoS One.

[21] Lee HS, Goh MJ, Kim J, Choi TJ, Kwang Lee H, Joo Na Y, Cho KH (2015). A systems-biological study on the identification of safe and effective molecular targets for the reduction of ultraviolet B-induced skin pigmentation. Sci Rep.

[22] Kim J, Park SM, Cho KH (2013). Discovery of a kernel for controlling biomolecular regulatory networks. Sci Rep.

[23] Wishart DS, Knox C, Guo AC, Cheng D, Shrivastava S, Tzur D, Gautam B, Hassanali M (2008). DrugBank: a knowledgebase for drugs, drug actions and drug targets. Nucleic Acids Res.

[24] Kim J, Vandamme D, Kim JR, Munoz AG, Kolch W, Cho KH (2014). Robustness and evolvability of the human signaling network. PLoS Comput Biol.

[25] Schlatter R, Schmich K, Avalos Vizcarra I, Scheurich P, Sauter T, Borner C, Ederer M, Merfort I, Sawodny O (2009). ON/OFF and beyond--a boolean model of apoptosis. PLoS Comput Biol.

[26] Oda K, Matsuoka Y, Funahashi A, Kitano H (2005). A comprehensive pathway map of epidermal growth factor receptor signaling. Mol Syst Biol.

[27] Calzone L, Gelay A, Zinovyev A, Radvanyi F, Barillot E (2008). A comprehensive modular map of molecular interactions in RB/E2F pathway. Mol Syst Biol.

[28] Cavallaro U, Christofori G (2004). Cell adhesion and signalling by cadherins and Ig-CAMs in cancer. Nat Rev Cancer.

[29] Bienz M, Clevers H (2000). Linking colorectal cancer to Wnt signaling. Cell.

[30] Polakis P (2000). Wnt signaling and cancer. Genes Dev.

[31] Birchmeier W, Behrens J (1994). Cadherin expression in carcinomas: role in the formation of cell junctions and the prevention of invasiveness. Biochim Biophys Acta.

[32] Perl AK, Wilgenbus P, Dahl U, Semb H, Christofori G (1998). A causal role for E-cadherin in the transition from adenoma to carcinoma. Nature.

[33] Tomar A, Schlaepfer DD (2009). Focal adhesion kinase: switching between GAPs and GEFs in the regulation of cell motility. Curr Opin Cell Biol.

[34] Musgrove EA, Caldon CE, Barraclough J, Stone A, Sutherland RL (2011). Cyclin D as a therapeutic target in cancer. Nat Rev Cancer.

[35] Fearon ER, Vogelstein B (1990). A genetic model for colorectal tumorigenesis. Cell.

[36] Conlin A, Smith G, Carey FA, Wolf CR, Steele RJ (2005). The prognostic significance of K-ras, p53, and APC mutations in colorectal carcinoma. Gut.

[37] Sartore-Bianchi A, Martini M, Molinari F, Veronese S, Nichelatti M, Artale S, Di Nicolantonio F, Saletti P, De Dosso S, Mazzucchelli L (2009). PIK3CA mutations in colorectal cancer are associated with clinical resistance to EGFR-targeted monoclonal antibodies. Cancer Res.

[38] Hsieh JS, Lin SR, Chang MY, Chen FM, Lu CY, Huang TJ, Huang YS, Huang CJ, Wang JY (2005). APC, K-ras, and p53 gene mutations in colorectal cancer patients: correlation to clinicopathologic features and postoperative surveillance. Am Surg.

[39] Phelps RA, Chidester S, Dehghanizadeh S, Phelps J, Sandoval IT, Rai K, Broadbent T, Sarkar S, Burt RW, Jones DA (2009). A two-step model for colon adenoma initiation and progression caused by APC loss. Cell.

[40] Kinch MS, Clark GJ, Der CJ, Burridge K (1995). Tyrosine phosphorylation regulates the adhesions of ras-transformed breast epithelia. J Cell Biol.

[41] Fodde R, Brabletz T (2007). Wnt/beta-catenin signaling in cancer stemness and malignant behavior. Curr Opin Cell Biol.

[42] Wood LD, Parsons DW, Jones S, Lin J, Sjoblom T, Leary RJ, Shen D, Boca SM, Barber T, Ptak J (2007). The genomic landscapes of human breast and colorectal cancers. Science.

[43] von Manstein V, Yang CM, Richter D, Delis N, Vafaizadeh V, Groner B (2013). Resistance of cancer cells to targeted therapies through the activation of compensating signaling loops. Curr Signal Transduct Ther.

[44] Wells A, Griffith L, Wells JZ, Taylor DP (2013). The dormancy dilemma: quiescence versus balanced proliferation. Cancer Res.

[45] Morin PJ (1999). beta-catenin signaling and cancer. Bioessays.

[46] Vigil D, Cherfils J, Rossman KL, Der CJ (2010). Ras superfamily GEFs and GAPs: validated and tractable targets for cancer therapy?. Nat Rev Cancer.

[47] Shen B, Estevez B, Xu Z, Kreutz B, Karginov A, Bai Y, Qian F, Norifumi U, Mosher D, Du X (2015). The interaction of Galpha13 with integrin beta1 mediates cell migration by dynamic regulation of RhoA. Mol Biol Cell.

[48] Brown JP, Taube C, Miyahara N, Koya T, Pelanda R, Gelfand EW, Torres RM (2007). Arhgef1 is required by T cells for the development of airway hyperreactivity and inflammation. Am J Respir Crit Care Med.

[49] Roberts PJ, Der CJ (2007). Targeting the Raf-MEK-ERK mitogen-activated protein kinase cascade for the treatment of cancer. Oncogene.

[50] Cristobal I, Manso R, Rincon R, Carames C, Senin C, Borrero A, Martinez-Useros J, Rodriguez M, Zazo S, Aguilera O (2014). PP2A inhibition is a common event in colorectal cancer and its restoration using FTY720 shows promising therapeutic potential. Mol Cancer Ther.

[51] Won JK, Yang HW, Shin SY, Lee JH, Heo WD, Cho KH (2012). The crossregulation between ERK and PI3K signaling pathways determines the tumoricidal efficacy of MEK inhibitor. J Mol Cell Biol.

[52] Cole SW, Sood AK (2012). Molecular pathways: beta-adrenergic signaling in cancer. Clin Cancer Res.

[53] Marinissen MJ, Gutkind JS (2001). G-protein-coupled receptors and signaling networks: emerging paradigms. Trends Pharmacol Sci.

[54] Luttrell LM, Ferguson SS, Daaka Y, Miller WE, Maudsley S, Della Rocca GJ, Lin F, Kawakatsu H, Owada K, Luttrell DK (1999). Beta-arrestin-dependent formation of beta2 adrenergic receptor-Src protein kinase complexes. Science.

[55] Akhurst RJ, Derynck R (2001). TGF-beta signaling in cancer--a double-edged sword. Trends Cell Biol.

[56] Kudo-Saito C, Shirako H, Takeuchi T, Kawakami Y (2009). Cancer metastasis is accelerated through immunosuppression during Snail-induced EMT of cancer cells. Cancer Cell.

[57] Lim SO, Kim H, Jung G (2010). p53 inhibits tumor cell invasion via the degradation of snail protein in hepatocellular carcinoma. FEBS Lett.

[58] Dow LE, O’Rourke KP, Simon J, Tschaharganeh DF, van Es JH, Clevers H, Lowe SW (2015). Apc restoration promotes cellular differentiation and reestablishes crypt homeostasis in colorectal cancer. Cell.

[59] McClellan JS, Dove C, Gentles AJ, Ryan CE, Majeti R (2015). Reprogramming of primary human Philadelphia chromosome-positive B cell acute lymphoblastic leukemia cells into nonleukemic macrophages. Proc Natl Acad Sci U S A.

[60] Mahalingam D, Kong CM, Lai J, Tay LL, Yang H, Wang X (2012). Reversal of aberrant cancer methylome and transcriptome upon direct reprogramming of lung cancer cells. Sci Rep.

[61] Powers S, Pollack RE (2016). Inducing stable reversion to achieve cancer control. Nat Rev Cancer.

[62] Du K, Tsichlis PN (2005). Regulation of the Akt kinase by interacting proteins. Oncogene.

[63] Roux PP, Blenis J (2004). ERK and p38 MAPK-activated protein kinases: a family of protein kinases with diverse biological functions. Microbiol Mol Biol Rev.

[64] Citri A, Yarden Y (2006). EGF-ERBB signalling: towards the systems level. Nat Rev Mol Cell Biol.

[65] Price LS, Leng J, Schwartz MA, Bokoch GM (1998). Activation of Rac and Cdc42 by integrins mediates cell spreading. Mol Biol Cell.

[66] Brakebusch C, Bouvard D, Stanchi F, Sakai T, Fassler R (2002). Integrins in invasive growth. J Clin Invest.

[67] Waring P, Mullbacher A (1999). Cell death induced by the Fas/Fas ligand pathway and its role in pathology. Immunol Cell Biol.

[68] Lamouille S, Xu J, Derynck R (2014). Molecular mechanisms of epithelial-mesenchymal transition. Nat Rev Mol Cell Biol.

[69] Takeda K, Matsuzawa A, Nishitoh H, Ichijo H (2003). Roles of MAPKKK ASK1 in stress-induced cell death. Cell Struct Funct.

[70] Hennessy BT, Smith DL, Ram PT, Lu Y, Mills GB (2005). Exploiting the PI3K/AKT pathway for cancer drug discovery. Nat Rev Drug Discov.

[71] Moon RT, Kohn AD, De Ferrari GV, Kaykas A (2004). WNT and beta-catenin signalling: diseases and therapies. Nat Rev Genet.

[72] Meek DW (2009). Tumour suppression by p53: a role for the DNA damage response?. Nat Rev Cancer.

[73] Bartek J, Lukas C, Lukas J (2004). Checking on DNA damage in S phase. Nat Rev Mol Cell Biol.

