# Supplementary material for: Attractor landscape analysis of colorectal tumorigenesis and its reversion
Source: BMC Syst Biol. 2016 Oct 20;10:96. doi: 10.1186/s12918-016-0341-9 (PMC5072344; doi:10.1186/s12918-016-0341-9)
Supplement: Additional file 4: — The Matlab and Python source codes for mathematical simulations. (ZIP 1070 kb) [file 12918_2016_341_MOESM4_ESM.zip › Source codes for attractor landscape analysis/Readme.docx]

**Source code for calculating attractors based on Boolean simulation.**

Usage:

Using Matlab 2014a in a Window Cluster system composed of 288 CPUs in parallel.

**Input:**

node_list_input.txt 🡪 Input node lists in network model

node_list_with_truthtable.txt 🡪 Node lists which have truthtable

truthtable_inputoutput 🡪 each file contains a logic table for individual signaling component. The file name of each file denotes a target molecule.

Node_name.input 🡪 Node lists to regulate the target node

Node_name.output 🡪 functional value of logical expressions

For example: truthtable of Casp3

| XIAP | Casp8 | Casp9 | Casp3 |
| --- | --- | --- | --- |
| 0 | 0 | 0 | 0 |
| 0 | 0 | 1 | 1 |
| 0 | 1 | 0 | 1 |
| 0 | 1 | 1 | 1 |
| 1 | 0 | 0 | 0 |
| 1 | 0 | 1 | 0 |
| 1 | 1 | 0 | 0 |
| 1 | 1 | 1 | 0 |

From above truth table, Casp3 is regulated by XIAP, Casp8 and Casp9 and the last column of table describes the activity state of the Casp3 for each regulating condition of the source nodes. This table can be converted in to input and output files:

Casp3.input 🡪 XIAP Casp8 Casp9

| 0 |
| --- |
| 1 |
| 1 |
| 1 |
| 0 |
| 0 |
| 0 |
| 0 |

Casp3.output 🡪

mutation_node_list.txt 🡪 Mutation sequence list for network model.

mutation_node_value.txt 🡪 Mutation state values for the mutation sequence list.

initial_state_list_condition.mat 🡪 Input node condition for simulation.

**Output :**

attractor_list{i} 🡪 Attractor list for initial state i.

**
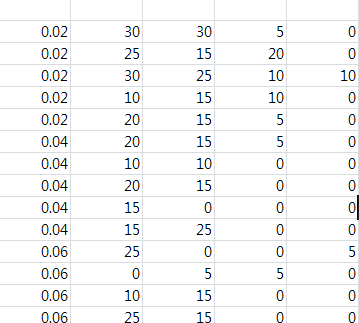

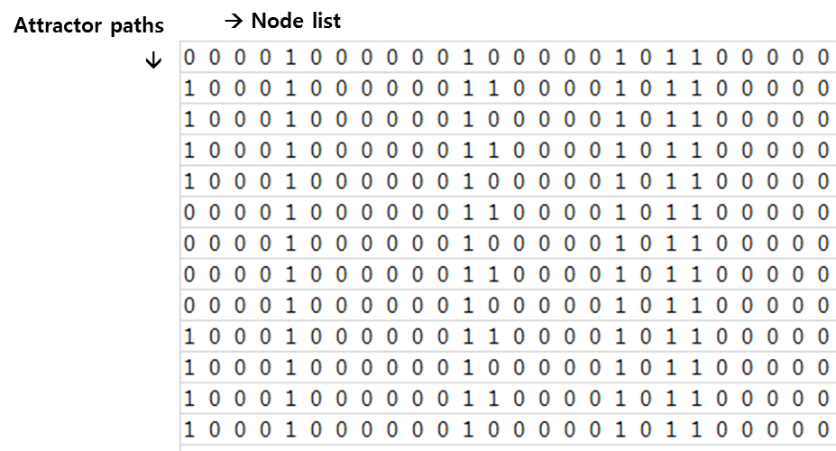
**

**Input intensity**

**🡪 Node list**

Copyright:

“Attractor Landscape Analysis of Colorectal Tumorigenesis and Its Reversion”

Sung-Hwan Cho, Sang-Min Park, Ho-Sung Lee, Hwang-Yeol Lee, and Kwang-Hyun Cho*

Laboratory for Systems Biology and Bio-Inspired Engineering, Department of Bio and Brain Engineering, Korea Advanced Institute of Science and Technology (KAIST), Republic of Korea.
