# Supplementary material for: Attractor landscape analysis of colorectal tumorigenesis and its reversion
Source: BMC Syst Biol. 2016 Oct 20;10:96. doi: 10.1186/s12918-016-0341-9 (PMC5072344; doi:10.1186/s12918-016-0341-9)
Supplement: Additional file 5: — Supporting Figures and Tables (contains 4 figures and 5 tables). (PDF 634 kb) [file 12918_2016_341_MOESM5_ESM.pdf]

Attractor Landscape Analysis of Colorectal Tumorigenesis and Its Reversion

Sung-Hwan Cho[1], Sang-Min Park[1], Ho-Sung Lee[1,2], Hwang-Yeol Lee[1], and Kwang-Hyun Cho[1,2,*]

[1]Laboratory for Systems Biology and Bio-Inspired Engineering, Department of Bio and Brain Engineering, Korea Advanced Institute of Science and Technology (KAIST), Daejeon, 34141, Republic of Korea

[2]Graduate School of Medical Science and Engineering, Korea Advanced Institute of Science and Technology (KAIST), Daejeon, 34141, Republic of Korea

Supporting Figures and Tables

[*]Corresponding author, E-mail: ckh@kaist.ac.kr, Phone: +82-42-350-4325, Fax: +82-42-350-4310, Web: http://sbie.kaist.ac.kr

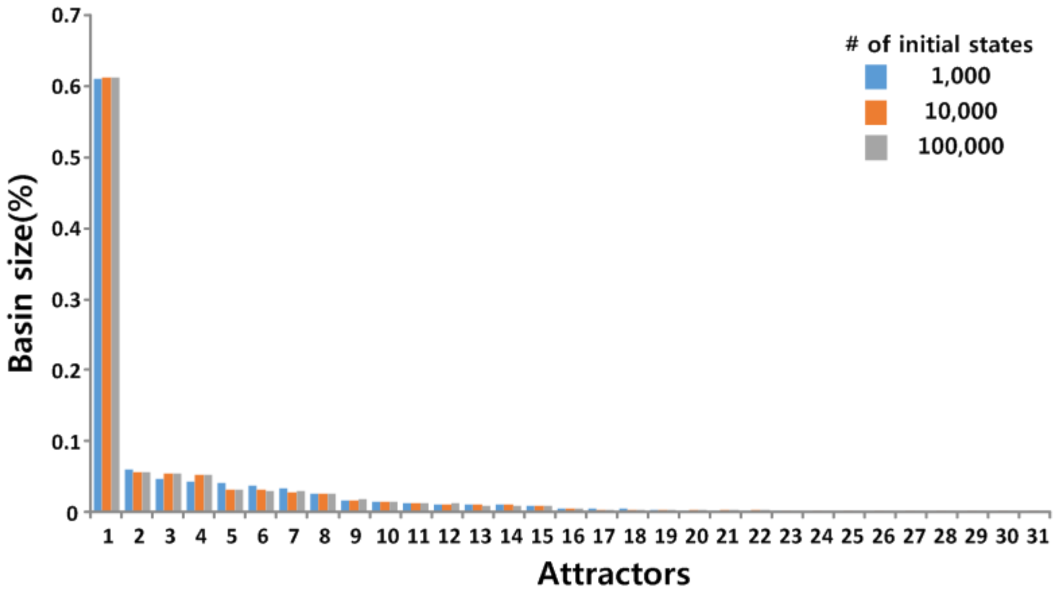

Figure S1. The ratio of basin sizes for different sampling numbers of initial states. The distribution of basin size is maintained regardless of sampling numbers of initial states.

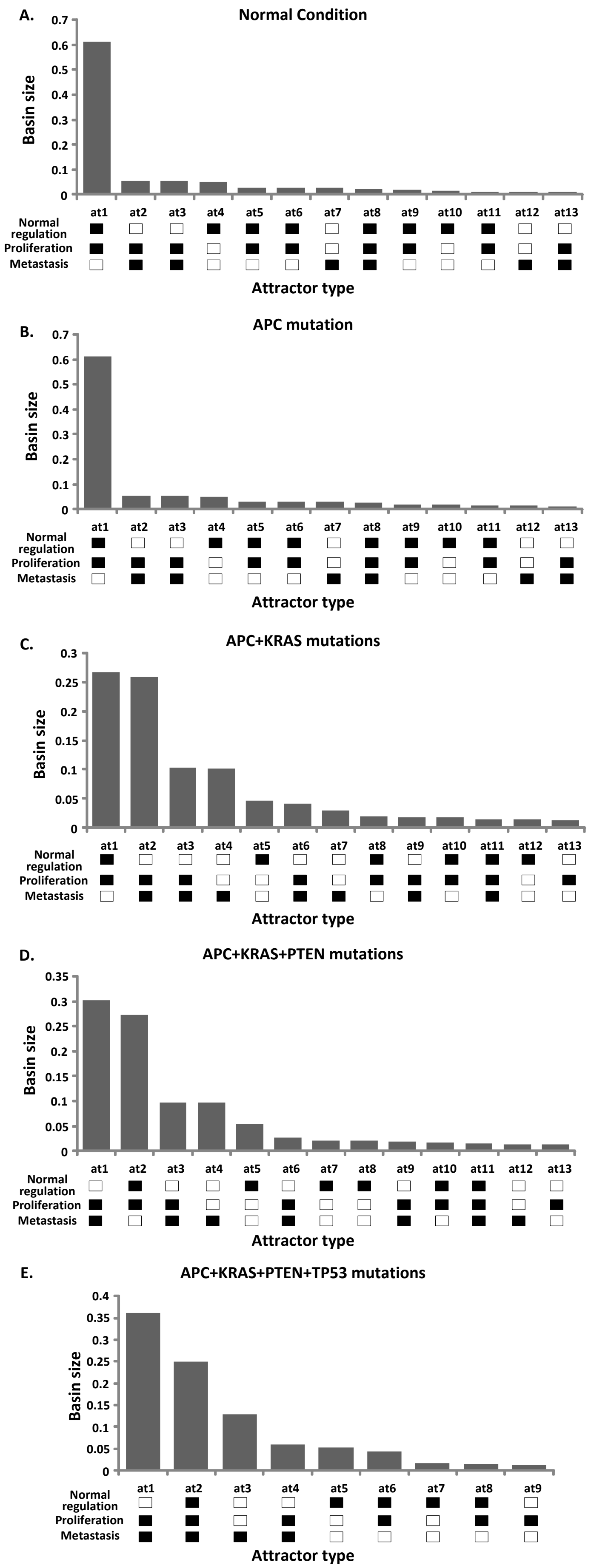
A.
Normal Condition
Basin size
0 0.1 0.2 0.3 0.4 0.5 0.6 0.7
at1 at2 at3 at4 at5 at6 at7 at8 at9 at10 at11 at12 at13
Normal regulation
Proliferation
Metastasis
Attractor type
B.
APC mutation
Basin size
0 0.1 0.2 0.3 0.4 0.5 0.6 0.7
at1 at2 at3 at4 at5 at6 at7 at8 at9 at10 at11 at12 at13
Normal regulation
Proliferation
Metastasis
Attractor type
C.
APC+KRAS mutations
Basin size
0 0.05 0.1 0.15 0.2 0.25 0.3
at1 at2 at3 at4 at5 at6 at7 at8 at9 at10 at11 at12 at13
Normal regulation
Proliferation
Metastasis
Attractor type
D.
APC+KRAS+PTEN mutations
Basin size
0 0.05 0.1 0.15 0.2 0.25 0.3 0.35
at1 at2 at3 at4 at5 at6 at7 at8 at9 at10 at11 at12 at13
Normal regulation
Proliferation
Metastasis
Attractor type
E.
APC+KRAS+PTEN+TP53 mutations
Basin size
0 0.05 0.1 0.15 0.2 0.25 0.3 0.35 0.4
at1 at2 at3 at4 at5 at6 at7 at8 at9
Normal regulation
Proliferation
Metastasis
Attractor type

Figure S2. The attractor classification according to three criteria and the distribution of basin size for attractors in each accumulation level of driver mutations. (A) Network in normal condition. (B) Network with APC mutation. (C) Network with APC and KRAS mutations. (D) Network with APC, KRAS and PTEN mutations. (E) Network with APC, KRAS, PTEN and TP53 mutations. White and black boxes represent cell phenotype as “off” or “on,” respectively.

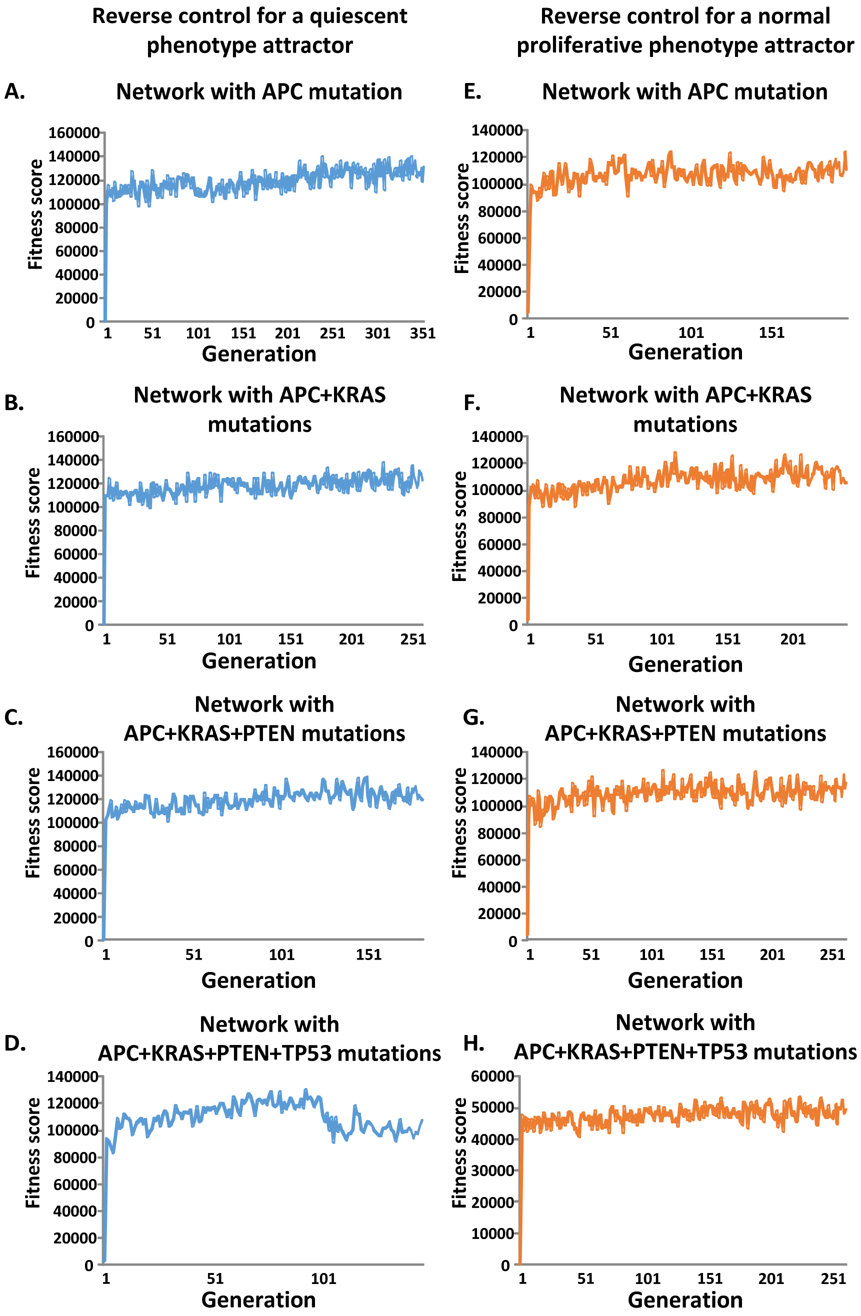

Figure S3. Trajectories of fitness score during the optimization process of a genetic algorithm for the identification of control nodes in each accumulation stage of driver mutations. (A, B, C, and D) The trajectories of fitness score in the case of controlling attractor landscape into a quiescent phenotype attractor. (A) Network with APC mutation. (B) Network with APC and KRAS mutations. (C) Network with APC, KRAS and PTEN mutations. (D) Network with APC, KRAS, PTEN and TP53 mutations. (E, F, G, and H) The trajectories of fitness score in the case of controlling attractor landscape into normal proliferative phenotype attractor. (E) Network with APC mutation. (F) Network with APC and KRAS mutations. (G) Network with APC, KRAS and PTEN mutations. (H) Network with APC, KRAS, PTEN and TP53 mutations.

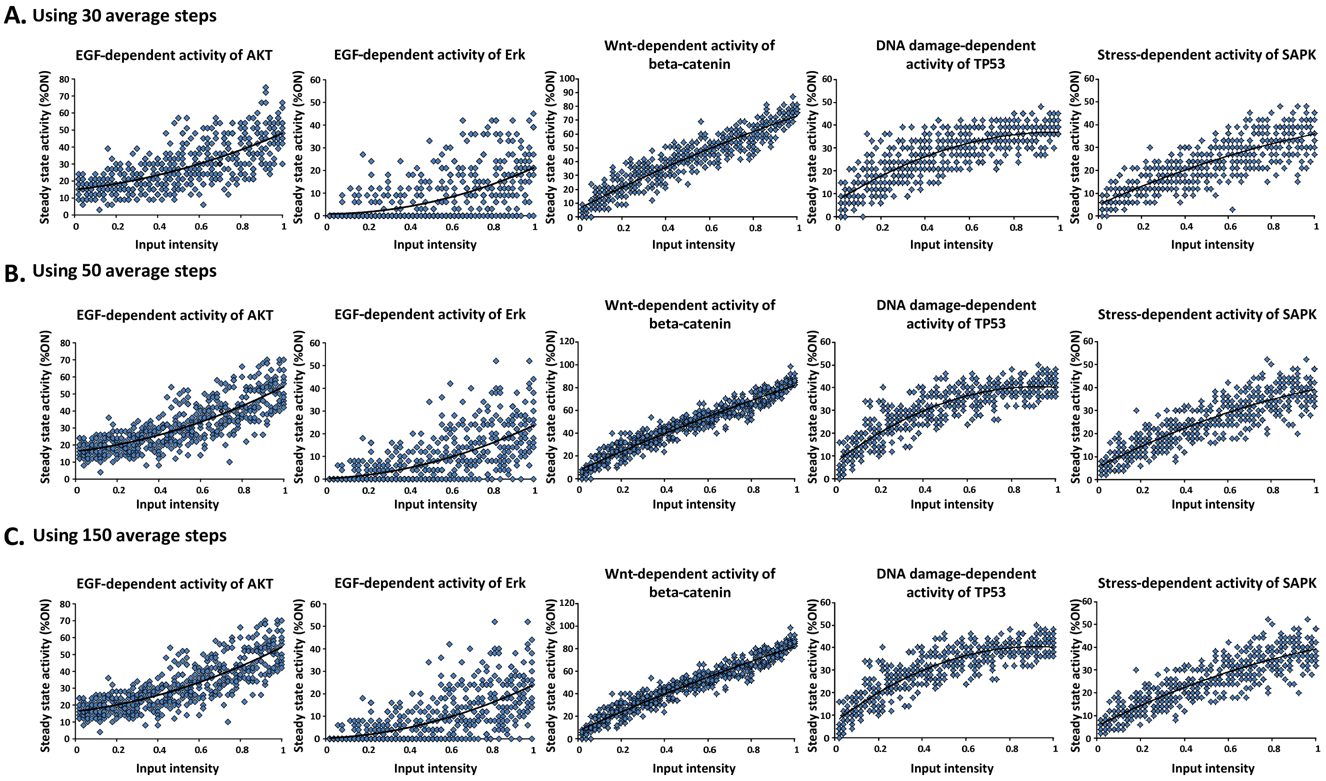

Figure S4. Qualitative input–output relationships in the Boolean model of human signaling network. (A) Using 30 average steps for the activity calculation. (B) Using 50 average steps for the activity calculation. (C) Using 150 average steps for the activity calculation.

Table S1. The stable states of the human signaling network in normal condition.

Nodes	Cyclic attractor-1																				Basin size : 0.6148																			
Rho	0	1	0	1	0	1	0	1	0	1	0	1	0	1	0	1	0	1	0	1	0	1	0	1	0	1	0	1	0	1	0	1	0	1	0	1	0	1	0	1
CyclinD	0	1	1	1	1	0	0	0	0	1	1	1	1	0	0	0	0	1	1	1	1	0	0	0	0	1	1	1	1	0	0	0	0	1	1	1	1	0	0	0
CyclinE	1	0	0	0	1	1	0	0	0	1	1	0	0	0	1	1	0	0	0	1	1	0	0	0	1	1	0	0	0	1	1	0	0	0	1	1	0	0	0	1
CyclinA	1	1	0	0	1	1	1	0	0	1	1	1	0	0	1	1	1	0	0	1	1	1	0	0	1	1	1	0	0	1	1	1	0	0	1	1	1	0	0	1
CyclinB	1	1	0	0	0	1	1	0	0	0	1	1	0	0	0	1	1	0	0	0	1	1	0	0	0	1	1	0	0	0	1	1	0	0	0	1	1	0	0	0
Casp-3	0	0	0	0	0	0	0	0	0	0	0	0	0	0	0	0	0	0	0	0	0	0	0	0	0	0	0	0	0	0	0	0	0	0	0	0	0	0	0	0
MMP	0	0	0	0	0	0	0	0	0	0	0	0	0	0	0	0	0	0	0	0	0	0	0	0	0	0	0	0	0	0	0	0	0	0	0	0	0	0	0	0
E-cad	1	1	1	1	1	1	1	1	1	1	1	1	1	1	1	1	1	1	1	1	1	1	1	1	1	1	1	1	1	1	1	1	1	1	1	1	1	1	1	1

Nodes	Cyclic attractor-2																				Basin size : 0.054																			
Rho	0	1	0	1	0	1	0	1	0	1	0	1	0	1	0	1	0	1	0	1	0	1	0	1	0	1	0	1	0	1	0	1	0	1	0	1	0	1	0	1
CyclinD	1	1	1	1	1	1	1	1	1	1	1	1	1	1	1	1	1	1	1	1	1	1	1	1	1	1	1	1	1	1	1	1	1	1	1	1	1	1	1	1
CyclinE	0	1	1	0	0	0	1	1	0	0	0	1	1	0	0	0	1	1	0	0	0	1	1	0	0	0	1	1	0	0	0	1	1	0	0	0	1	1	0	0
CyclinA	0	1	1	1	0	0	1	1	1	0	0	1	1	1	0	0	1	1	1	0	0	1	1	1	0	0	1	1	1	0	0	1	1	1	0	0	1	1	1	0
CyclinB	0	0	1	1	0	0	0	1	1	0	0	0	1	1	0	0	0	1	1	0	0	0	1	1	0	0	0	1	1	0	0	0	1	1	0	0	0	1	1	0
Casp-3	0	0	0	0	0	0	0	0	0	0	0	0	0	0	0	0	0	0	0	0	0	0	0	0	0	0	0	0	0	0	0	0	0	0	0	0	0	0	0	0
MMP	1	1	1	1	1	1	1	1	1	1	1	1	1	1	1	1	1	1	1	1	1	1	1	1	1	1	1	1	1	1	1	1	1	1	1	1	1	1	1	1
E-cad	0	0	0	0	0	0	0	0	0	0	0	0	0	0	0	0	0	0	0	0	0	0	0	0	0	0	0	0	0	0	0	0	0	0	0	0	0	0	0	0

Nodes	Cyclic attractor-3																				Basin size : 0.0533									
Rho	1	0	1	0	0	1	1	0	1	0	1	0	1	1	0	1	0	1	0	0	1	1	0	1	0	1	0	1	1	0
CyclinD	1	1	1	1	1	1	1	1	1	1	1	1	1	1	1	1	1	1	1	1	1	1	1	1	1	1	1	1	1	1
CyclinE	0	0	0	1	0	0	0	0	1	0	0	0	0	1	0	0	0	0	1	0	0	0	0	1	0	0	0	0	1	0
CyclinA	0	0	0	1	1	0	0	0	1	1	0	0	0	1	1	0	0	0	1	1	0	0	0	1	1	0	0	0	1	1
CyclinB	1	0	0	0	1	1	0	0	0	1	1	0	0	0	1	1	0	0	0	1	1	0	0	0	1	1	0	0	0	1
Casp-3	0	0	0	0	0	0	0	0	0	0	0	0	0	0	0	0	0	0	0	0	0	0	0	0	0	0	0	0	0	0
MMP	1	1	1	1	1	1	1	1	1	1	1	1	1	1	1	1	1	1	1	1	1	1	1	1	1	1	1	1	1	1
E-cad	0	0	0	0	0	0	0	0	0	0	0	0	0	0	0	0	0	0	0	0	0	0	0	0	0	0	0	0	0	0

Nodes	Cyclic attractor-4																				Basin size : 0.0512																			
Rho	0	0	0	0	0	0	0	0	0	0	0	0	0	0	0	0	0	0	0	0	0	0	0	0	0	0	0	0	0	0	0	0	0	0	0	0	0	0	0	0
CyclinD	0	0	0	0	0	0	0	0	0	0	0	0	0	0	0	0	0	0	0	0	0	0	0	0	0	0	0	0	0	0	0	0	0	0	0	0	0	0	0	0
CyclinE	0	0	1	0	1	0	0	0	0	0	1	0	0	0	0	0	1	0	1	0	0	0	0	0	1	0	0	0	0	0	1	0	1	0	0	0	0	0	1	0
CyclinA	0	0	0	0	1	1	1	1	0	0	1	1	1	1	0	0	0	0	1	1	1	1	0	0	1	1	1	1	0	0	0	0	1	1	1	1	0	0	1	1
CyclinB	0	1	0	0	0	0	1	1	0	0	0	0	1	0	0	1	0	0	0	0	1	1	0	0	0	0	1	0	0	1	0	0	0	0	1	1	0	0	0	0
Casp-3	0	0	0	0	0	0	0	0	0	0	0	0	0	0	0	0	0	0	0	0	0	0	0	0	0	0	0	0	0	0	0	0	0	0	0	0	0	0	0	0
MMP	0	0	0	0	0	0	0	0	0	0	0	0	0	0	0	0	0	0	0	0	0	0	0	0	0	0	0	0	0	0	0	0	0	0	0	0	0	0	0	0
E-cad	1	1	1	1	1	1	1	1	1	1	1	1	1	1	1	1	1	1	1	1	1	1	1	1	1	1	1	1	1	1	1	1	1	1	1	1	1	1	1	1

Nodes	Cyclic attractor-5																				Basin size : 0.0288																			
Rho	0	1	0	1	0	1	0	1	0	1	0	1	0	1	0	1	0	1	0	1	0	1	0	1	0	1	0	1	0	1	0	1	0	1	0	1	0	1	0	1
CyclinD	0	1	1	1	1	0	0	0	0	1	1	1	1	0	0	0	0	1	1	1	1	0	0	0	0	1	1	1	1	0	0	0	0	1	1	1	1	0	0	0
CyclinE	1	1	0	0	0	1	1	0	0	0	1	1	0	0	0	1	1	0	0	0	1	1	0	0	0	1	1	0	0	0	1	1	0	0	0	1	1	0	0	0
CyclinA	1	1	1	0	0	1	1	1	0	0	1	1	1	0	0	1	1	1	0	0	1	1	1	0	0	1	1	1	0	0	1	1	1	0	0	1	1	1	0	0
CyclinB	0	1	1	0	0	0	1	1	0	0	0	1	1	0	0	0	1	1	0	0	0	1	1	0	0	0	1	1	0	0	0	1	1	0	0	0	1	1	0	0
Casp-3	0	0	0	0	0	0	0	0	0	0	0	0	0	0	0	0	0	0	0	0	0	0	0	0	0	0	0	0	0	0	0	0	0	0	0	0	0	0	0	0
MMP	1	1	1	1	1	1	1	1	1	1	1	1	1	1	1	1	1	1	1	1	1	1	1	1	1	1	1	1	1	1	1	1	1	1	1	1	1	1	1	1
E-cad	1	1	1	1	1	1	1	1	1	1	1	1	1	1	1	1	1	1	1	1	1	1	1	1	1	1	1	1	1	1	1	1	1	1	1	1	1	1	1	1

Nodes	Cyclic attractor-6																				Basin size : 0.0287									
Rho	0	1	1	0	1	0	1	0	1	1	0	1	0	1	0	0	1	1	0	1	0	1	0	1	1	0	1	0	1	0
CyclinD	1	1	1	1	1	1	1	1	1	1	1	1	1	1	1	1	1	1	1	1	1	1	1	1	1	1	1	1	1	1
CyclinE	1	0	0	0	1	1	0	0	0	1	1	0	0	0	1	1	0	0	0	1	1	0	0	0	1	1	0	0	0	1
CyclinA	0	0	0	0	0	0	0	0	0	0	0	0	0	0	0	0	0	0	0	0	0	0	0	0	0	0	0	0	0	0
CyclinB	1	0	0	0	1	1	0	0	0	1	1	0	0	0	1	1	0	0	0	1	1	0	0	0	1	1	0	0	0	1
Casp-3	0	0	0	0	0	0	0	0	0	0	0	0	0	0	0	0	0	0	0	0	0	0	0	0	0	0	0	0	0	0
MMP	1	1	1	1	1	1	1	1	1	1	1	1	1	1	1	1	1	1	1	1	1	1	1	1	1	1	1	1	1	1
E-cad	0	0	0	0	0	0	0	0	0	0	0	0	0	0	0	0	0	0	0	0	0	0	0	0	0	0	0	0	0	0

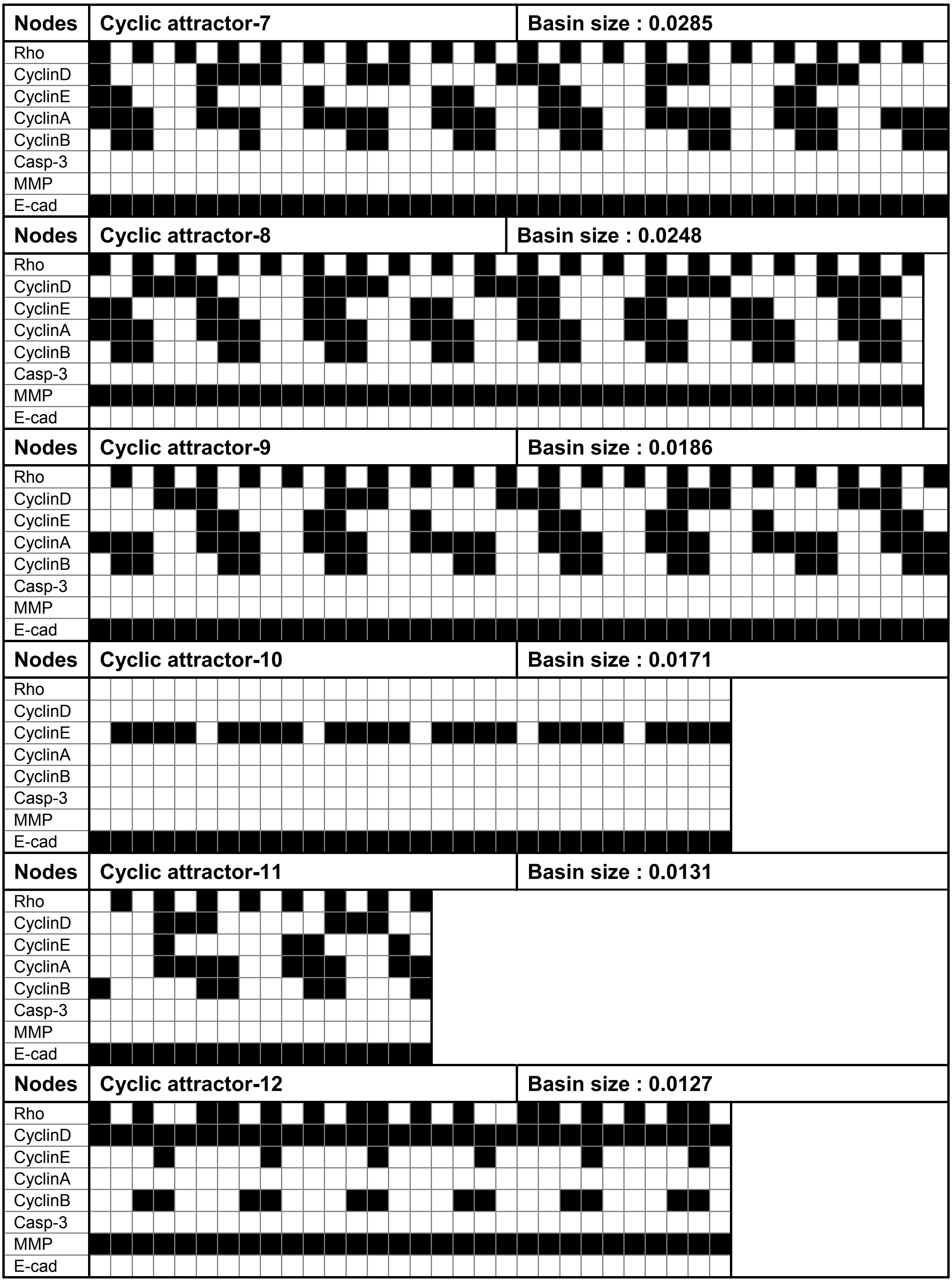
Nodes
Cyclic attractor-7
Basin size : 0.0285
Rho
CyclinD
CyclinE
CyclinA
CyclinB
Casp-3
MMP
E-cad
Nodes
Cyclic attractor-8
Basin size : 0.0248
Rho
CyclinD
CyclinE
CyclinA
CyclinB
Casp-3
MMP
E-cad
Nodes
Cyclic attractor-9
Basin size : 0.0186
Rho
CyclinD
CyclinE
CyclinA
CyclinB
Casp-3
MMP
E-cad
Nodes
Cyclic attractor-10
Basin size : 0.0171
Rho
CyclinD
CyclinE
CyclinA
CyclinB
Casp-3
MMP
E-cad
Nodes
Cyclic attractor-11
Basin size : 0.0131
Rho
CyclinD
CyclinE
CyclinA
CyclinB
Casp-3
MMP
E-cad
Nodes
Cyclic attractor-12
Basin size : 0.0127
Rho
CyclinD
CyclinE
CyclinA
CyclinB
Casp-3
MMP
E-cad

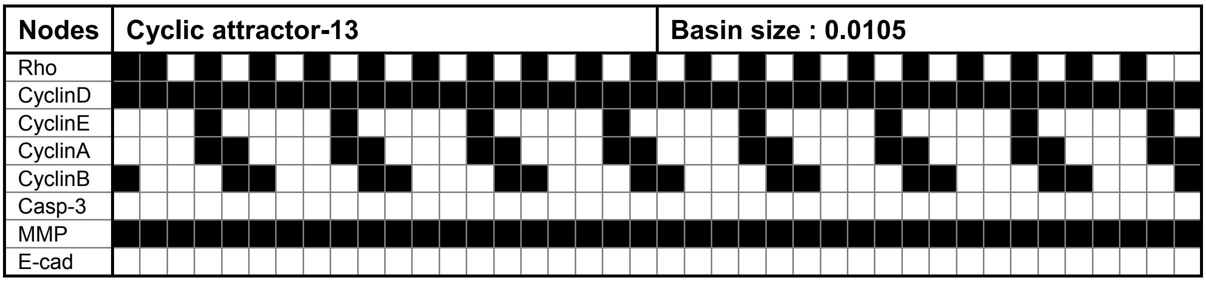
Nodes
Cyclic attractor-13
Basin size : 0.0105
Rho
CyclinD
CyclinE
CyclinA
CyclinB
Casp-3
MMP
E-cad

Table S2. The stable states of the human signaling network with APC mutation.

Nodes	Cyclic attractor-1	Basin size : 0.6147
Rho		
CyclinD		
CyclinE		
CyclinA		
CyclinB		
Casp-3		
MMP		
E-cad		

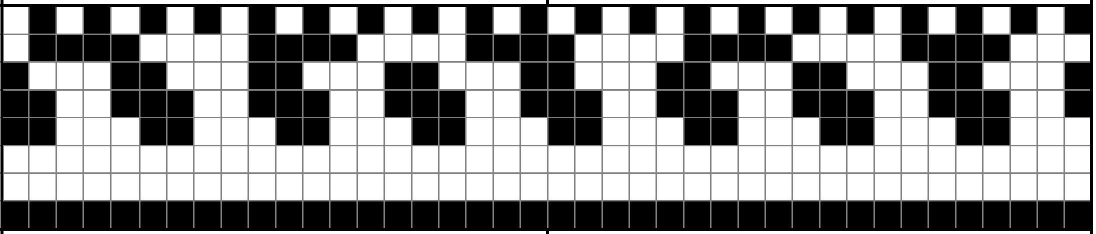

Nodes	Cyclic attractor-2	Basin size : 0.0542
Rho		
CyclinD		
CyclinE		
CyclinA		
CyclinB		
Casp-3		
MMP		
E-cad		

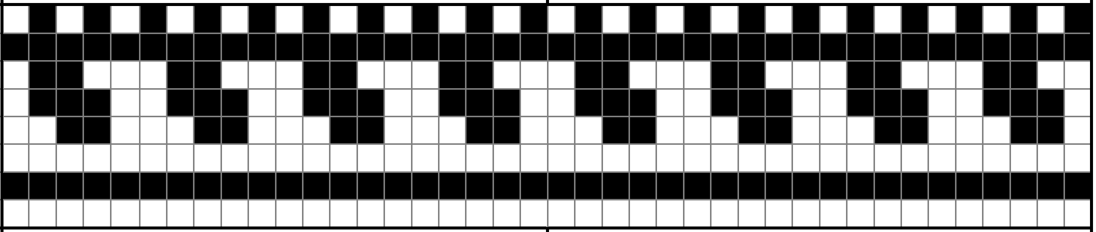

Nodes	Cyclic attractor-3	Basin size : 0.0535
Rho		
CyclinD		
CyclinE		
CyclinA		
CyclinB		
Casp-3		
MMP		
E-cad		

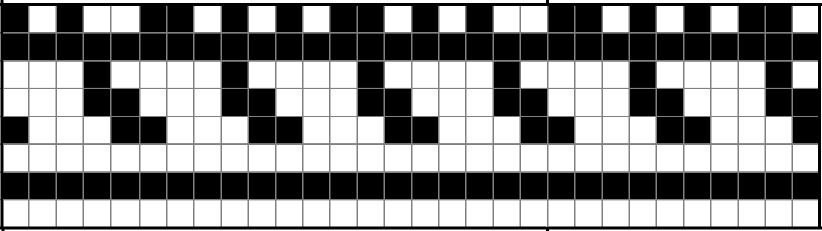

Nodes	Cyclic attractor-4	Basin size : 0.0511
Rho		
CyclinD		
CyclinE		
CyclinA		
CyclinB		
Casp-3		
MMP		
E-cad		

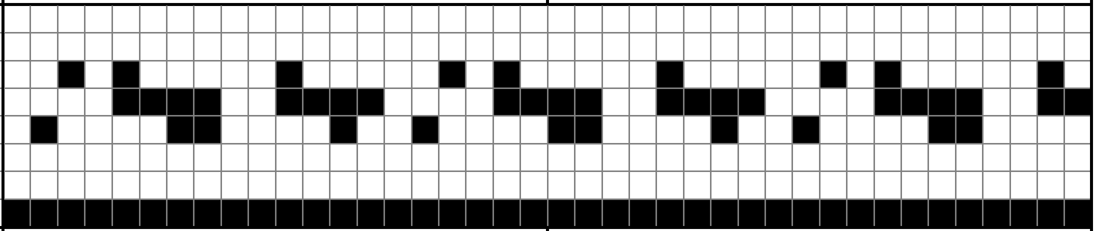

Nodes	Cyclic attractor-5	Basin size : 0.0288
Rho		
CyclinD		
CyclinE		
CyclinA		
CyclinB		
Casp-3		
MMP		
E-cad		

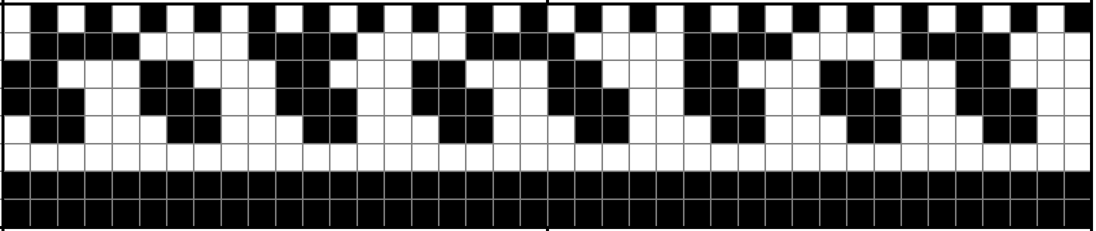

Nodes	Cyclic attractor-6	Basin size : 0.0285
Rho		
CyclinD		
CyclinE		
CyclinA		
CyclinB		
Casp-3		
MMP		
E-cad		

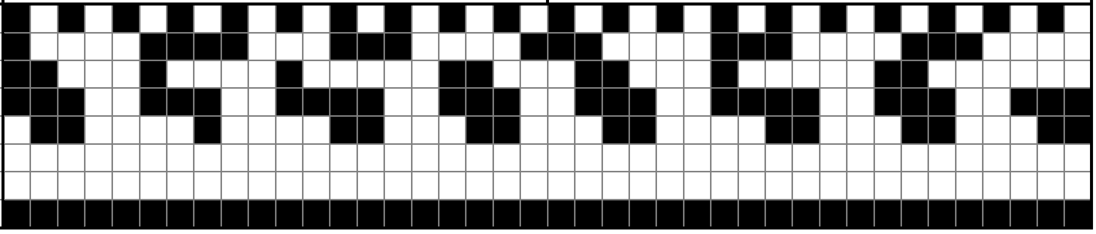

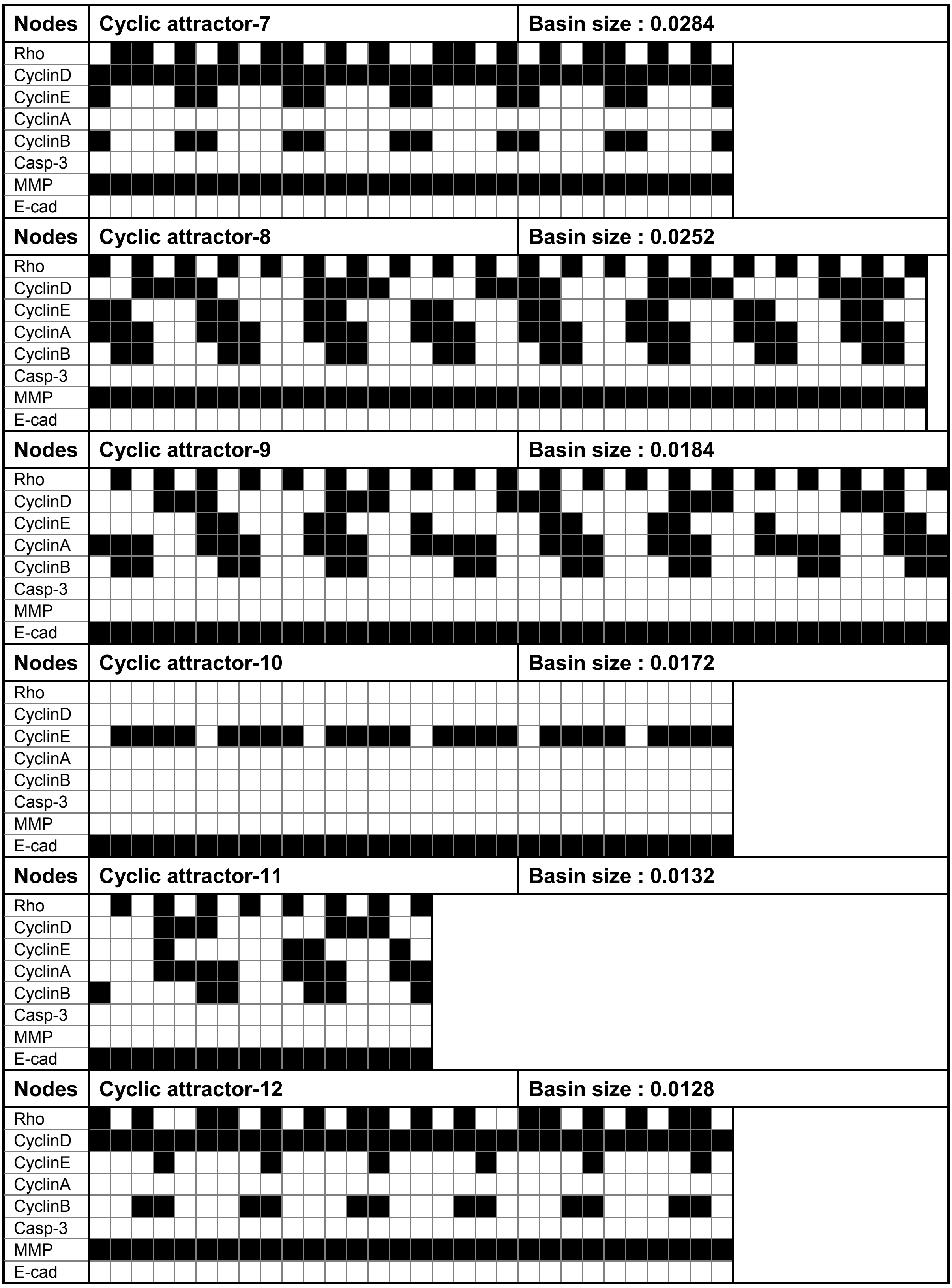
Nodes
Cyclic attractor-7
Basin size : 0.0284
Rho
CyclinD
CyclinE
CyclinA
CyclinB
Casp-3
MMP
E-cad
Nodes
Cyclic attractor-8
Basin size : 0.0252
Rho
CyclinD
CyclinE
CyclinA
CyclinB
Casp-3
MMP
E-cad
Nodes
Cyclic attractor-9
Basin size : 0.0184
Rho
CyclinD
CyclinE
CyclinA
CyclinB
Casp-3
MMP
E-cad
Nodes
Cyclic attractor-10
Basin size : 0.0172
Rho
CyclinD
CyclinE
CyclinA
CyclinB
Casp-3
MMP
E-cad
Nodes
Cyclic attractor-11
Basin size : 0.0132
Rho
CyclinD
CyclinE
CyclinA
CyclinB
Casp-3
MMP
E-cad
Nodes
Cyclic attractor-12
Basin size : 0.0128
Rho
CyclinD
CyclinE
CyclinA
CyclinB
Casp-3
MMP
E-cad

Nodes	Cyclic attractor-13																				Basin size : 0.0103																			
Rho	■	■		■		■		■		■		■		■		■		■		■		■		■		■		■		■		■		■		■		■		
CyclinD	■	■	■	■	■	■	■	■	■	■	■	■	■	■	■	■	■	■	■	■	■	■	■	■	■	■	■	■	■	■	■	■	■	■	■	■	■	■	■	■
CyclinE				■					■					■					■					■					■					■					■	
CyclinA				■	■				■	■				■	■				■	■				■	■				■	■				■	■				■	■
CyclinB	■				■	■				■	■				■	■				■	■				■	■				■	■				■	■				■
Casp-3																																								
MMP	■	■	■	■	■	■	■	■	■	■	■	■	■	■	■	■	■	■	■	■	■	■	■	■	■	■	■	■	■	■	■	■	■	■	■	■	■	■	■	■
E-cad																																								

Table S3. The stable states of the human signaling network with APC and KRAS mutations.

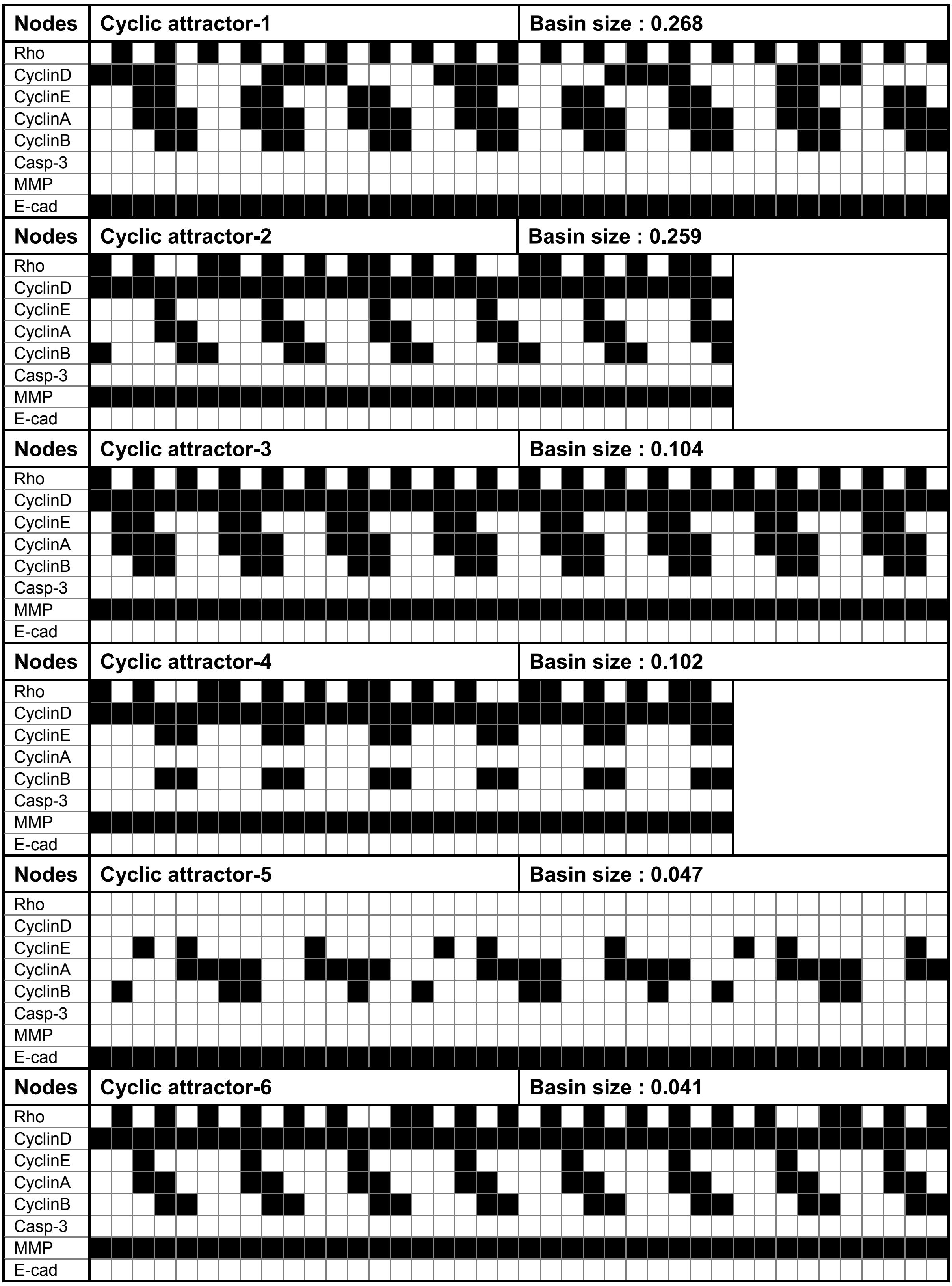

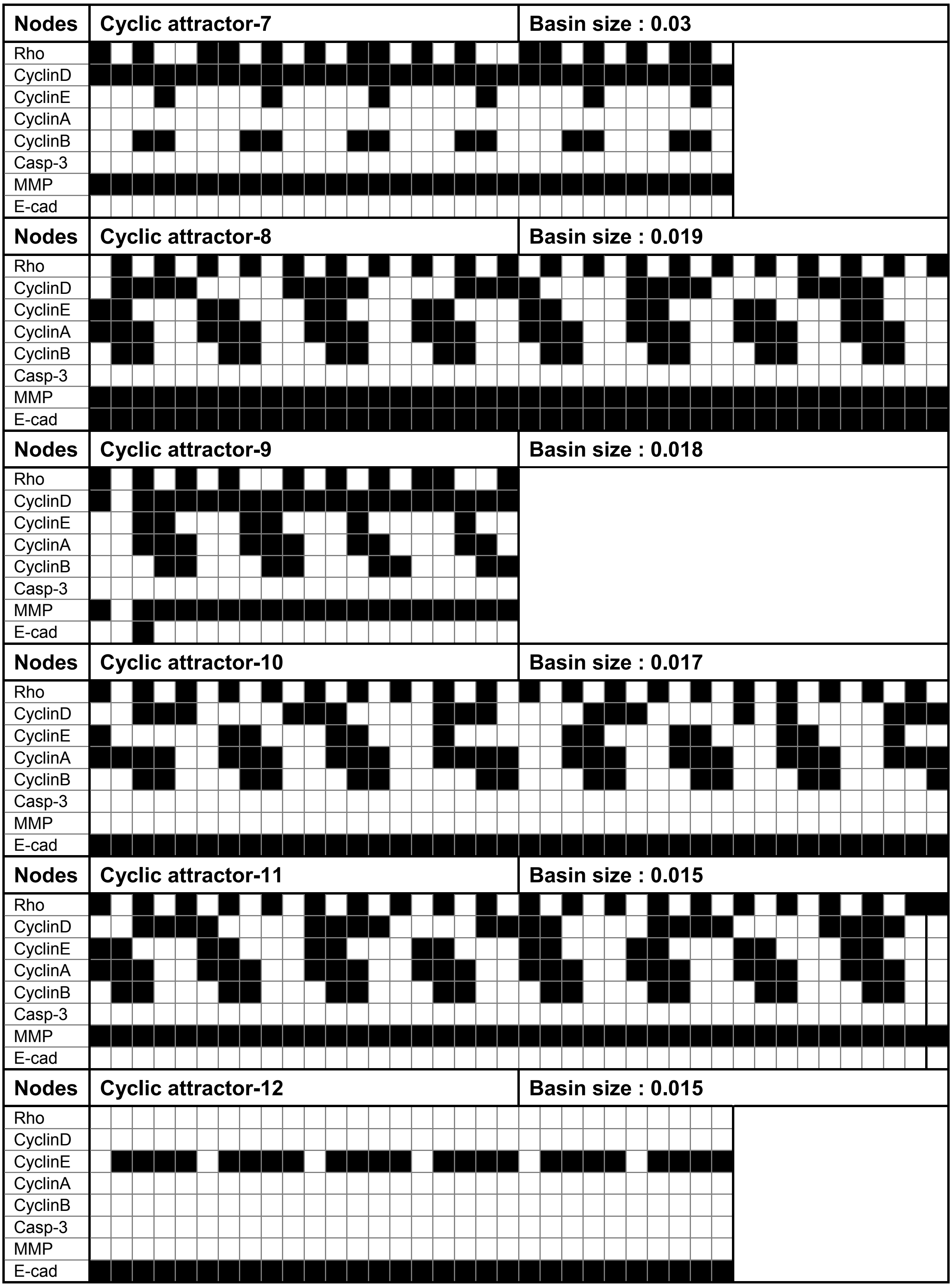
Nodes
Cyclic attractor-7
Basin size : 0.03
Cyclic attractor-8
Basin size : 0.019
Cyclic attractor-9
Basin size : 0.018
Cyclic attractor-10
Basin size : 0.017
Cyclic attractor-11
Basin size : 0.015
Cyclic attractor-12
Basin size : 0.015
Rho
CyclinD
CyclinE
CyclinA
CyclinB
Casp-3
MMP
E-cad

Nodes	Cyclic attractor-13	Basin size : 0.012
Rho		
CyclinD		
CyclinE		
CyclinA		
CyclinB		
Casp-3		
MMP		
E-cad		

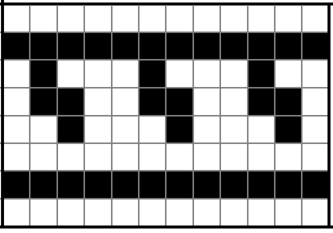

Table S4. The stable states of the human signaling network with APC, KRAS and PTEN mutations.

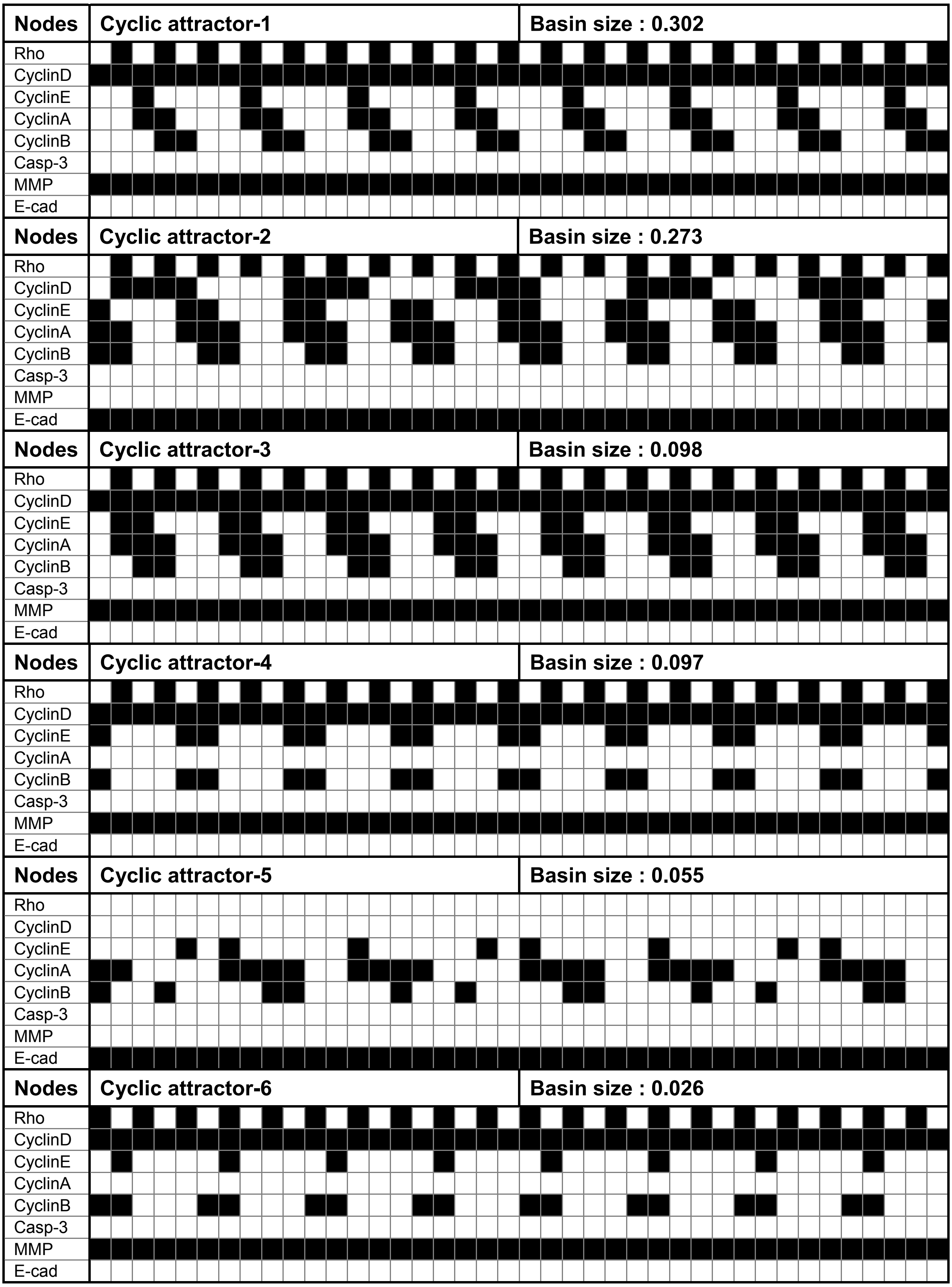

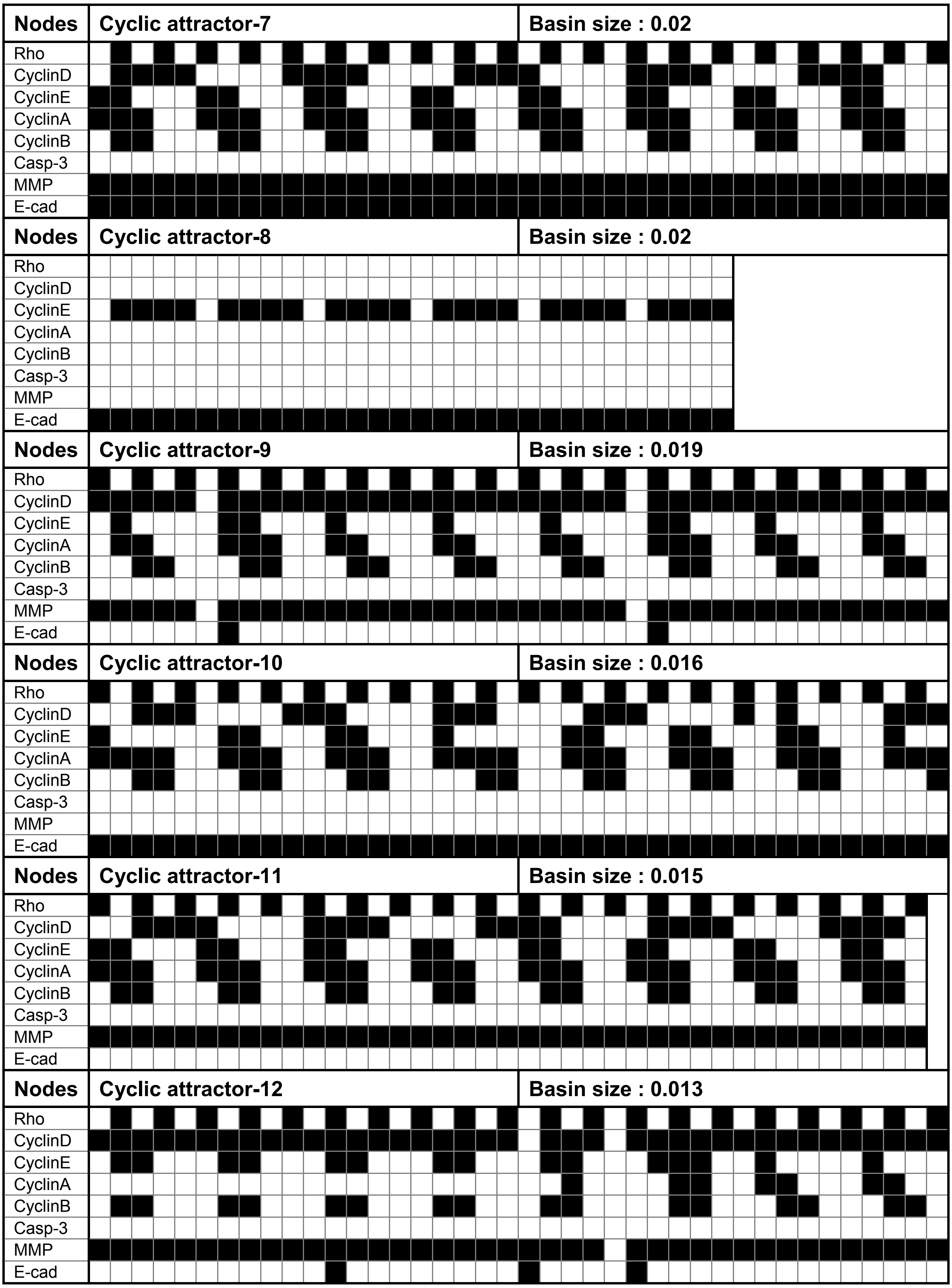
Nodes
Cyclic attractor-7
Basin size : 0.02
Rho
CyclinD
CyclinE
CyclinA
CyclinB
Casp-3
MMP
E-cad
Nodes
Cyclic attractor-8
Basin size : 0.02
Rho
CyclinD
CyclinE
CyclinA
CyclinB
Casp-3
MMP
E-cad
Nodes
Cyclic attractor-9
Basin size : 0.019
Rho
CyclinD
CyclinE
CyclinA
CyclinB
Casp-3
MMP
E-cad
Nodes
Cyclic attractor-10
Basin size : 0.016
Rho
CyclinD
CyclinE
CyclinA
CyclinB
Casp-3
MMP
E-cad
Nodes
Cyclic attractor-11
Basin size : 0.015
Rho
CyclinD
CyclinE
CyclinA
CyclinB
Casp-3
MMP
E-cad
Nodes
Cyclic attractor-12
Basin size : 0.013
Rho
CyclinD
CyclinE
CyclinA
CyclinB
Casp-3
MMP
E-cad

Nodes	Cyclic attractor-13	Basin size : 0.012
Rho		
CyclinD		
CyclinE		
CyclinA		
CyclinB		
Casp-3		
MMP		
E-cad		

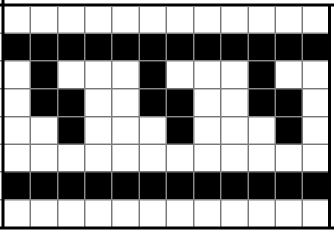

Table S5. The stable states of the human signaling network with APC, KRAS, PTEN and TP53 mutations.

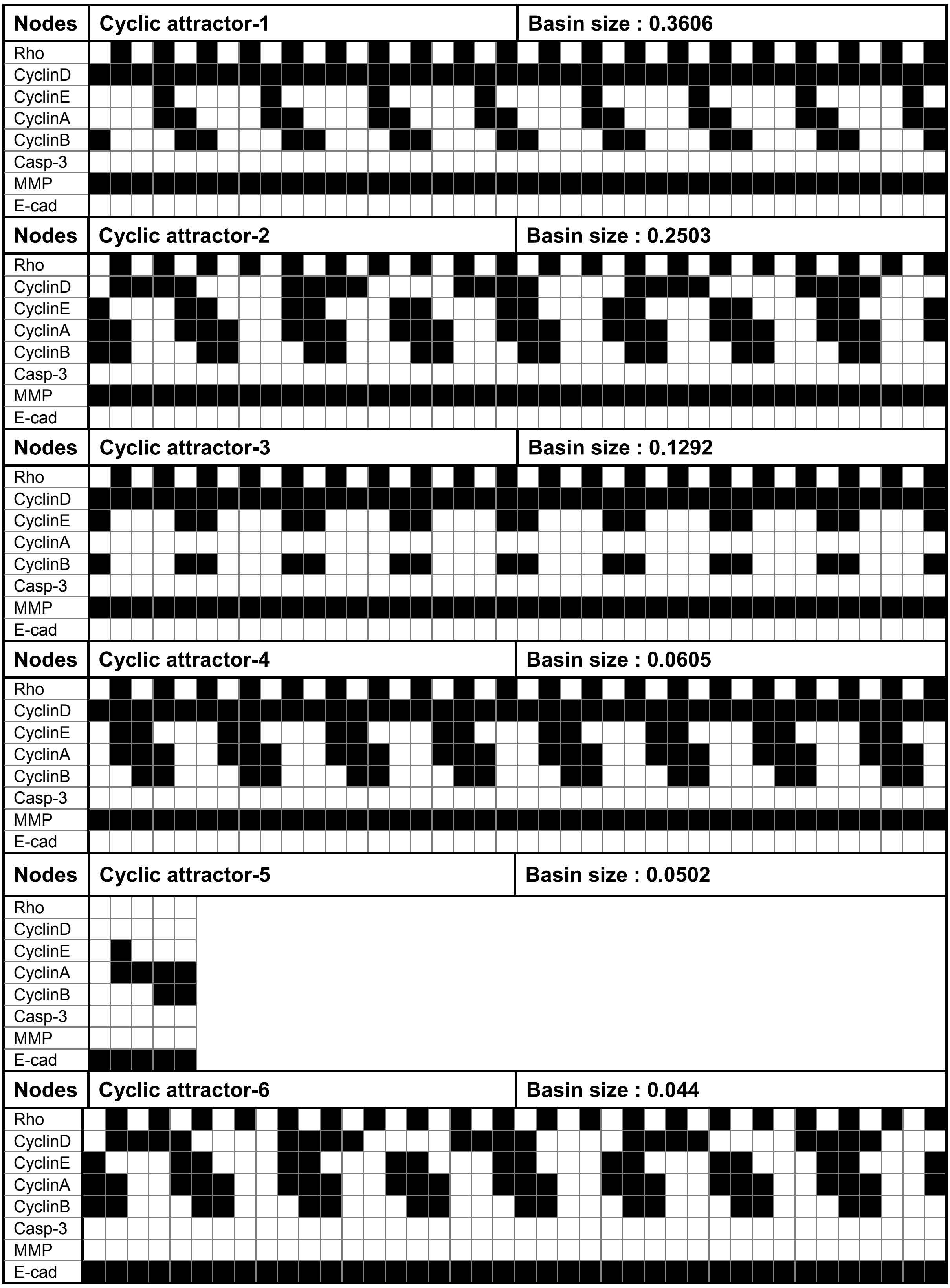

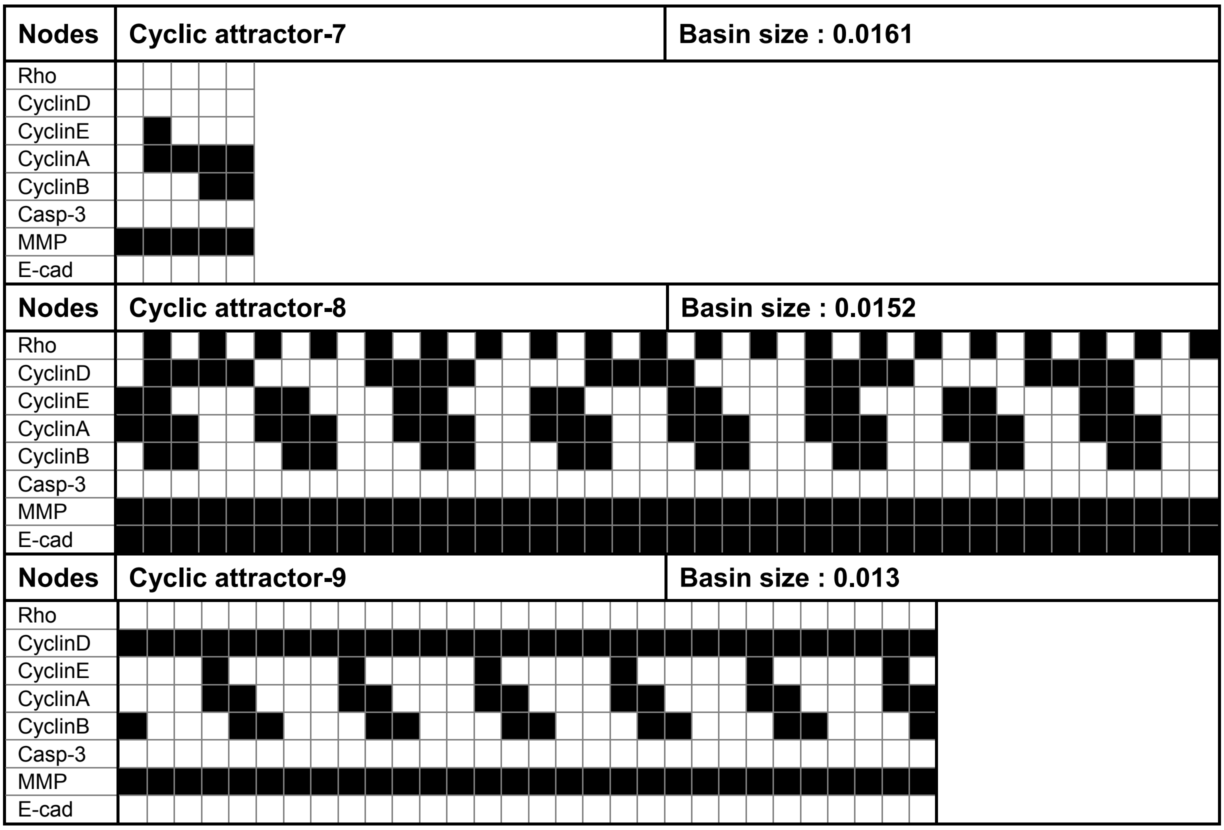
Nodes
Cyclic attractor-7
Basin size : 0.0161
Rho
CyclinD
CyclinE
CyclinA
CyclinB
Casp-3
MMP
E-cad
Nodes
Cyclic attractor-8
Basin size : 0.0152
Rho
CyclinD
CyclinE
CyclinA
CyclinB
Casp-3
MMP
E-cad
Nodes
Cyclic attractor-9
Basin size : 0.013
Rho
CyclinD
CyclinE
CyclinA
CyclinB
Casp-3
MMP
E-cad
